# Airway stenosis: classification, pathogenesis, and clinical management

**DOI:** 10.1002/mco2.70076

**Published:** 2025-01-26

**Authors:** Pengwei Zhao, Zheng Jiang, Xuexin Li, Mailudan Ainiwaer, Leyu Li, Dejuan Wang, Lixiao Fan, Fei Chen, Jun Liu

**Affiliations:** ^1^ Department of Otolaryngology ‐ Head & Neck Surgery West China Hospital Sichuan University Chengdu Sichuan China; ^2^ Department of Otolaryngology ‐ Head & Neck Surgery Head and Neck Surgical Center West China Hospital Sichuan University Chengdu Sichuan China; ^3^ Department of Otolaryngology Head and Neck Surgery Qilu Hospital (Qingdao) Cheeloo College of Medicine Shandong University Qingdao Shandong China

**Keywords:** airway stenosis, clinical management, laryngotracheal stenosis, pathogenesis, targeted therapy

## Abstract

Airway stenosis (AS) is a fibroinflammatory disease characterized by abnormal activation of fibroblasts and excessive synthesis of extracellular matrix, which has puzzled many doctors despite its relatively low prevalence. Traditional treatment such as endoscopic surgery, open surgery, and adjuvant therapy have many disadvantages and are limited in the treatment of patients with recurrent AS. Therefore, it is urgent to reveal the pathogenesis of AS and accelerate its clinical transformation. Based on the discovered pathogenesis, including fibrosis, inflammation, epithelial–mesenchymal transition, metabolic reprogramming, microbiome, genetic susceptibility, and other mechanisms, researchers have developed a series of treatments, such as drug therapy, gene therapy, stem cell therapy, growth factor therapy, protein therapy, and photodynamic therapy. This review introduces the classification of AS, explores the existing pathogenesis and preclinical treatments developed based on the pathogenesis, and finally summarizes the current clinical management. In addition, the prospect of exploring the interaction between different types of cells and between microorganisms and cells to identify the intersection of multiple mechanisms based on single‐cell RNA sequencing, 16S rRNA gene sequencing and shotgun metagenomic sequencing is worth looking forward to.

## Graphical abstract

The development of AS can be affected by many factors, and fibrosis is the core process. Inflammation, Epithelial–mesenchymal transition and metabolic reprogramming can accelerate the development of AS by promoting the process of fibrosis. And the rest also show the correlation with AS. Targeting these aberrant signaling pathways and molecular mechanism can lead to development of effective treatment of AS.

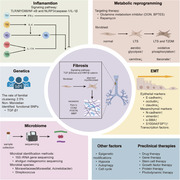

## 1 INTRODUCTION

Airway stenosis (AS) is a condition characterized by the narrowing of any part of the respiratory tract, especially supraglottic, glottic, subglottic, and tracheal stenosis, which are collectively referred to as laryngotracheal stenosis (LTS).^1^ LTS is the primary focus of researchers aiming to alleviate AS, and a significant amount of preclinical basic research is dedicated to this field. LTS is caused by various factors, including iatrogenic, traumatic, idiopathic, and congenital origins. The incidence rate of LTS is approximately 0.2–0.71 per 100,000 persons per year, with prolonged intubation and tracheostomy being the most common causes of iatrogenic LTS.^2^, ^3^, ^4^ Low incidence rate does not mean that the disease is not serious. On the contrary, LTS, one of the world's difficult fibrosis diseases, has puzzled many doctors, especially otolaryngologists. Risk factors include intubation, infections, obesity, diabetes, and gastroesophageal reflux disease.^5^ Traditional treatments mainly include endoscopic surgery, open surgery, and adjuvant therapy. Although endoscopic surgery can better protect the patient's swallowing and voice function, due to its high recurrence rate, open surgery has become the “gold standard” for the treatment of AS.^6^ In contrast, the drug treatment options for AS are quite limited. Although mitomycin, steroids, and some biological agents have shown certain efficacy, there is currently no specific drug.^7^, ^8^, ^9^ Despite these treatments, therapeutic outcomes remain suboptimal, with over 50% of patients, particularly those with iatrogenic etiologies, remaining tracheostomy dependent.^4^

The occurrence and development mechanism of AS is not yet fully understood, and researchers have long been committed to exploring its molecular mechanisms and signaling pathways in order to provide a theoretical basis for new treatments. Although drug targeted therapy, gene therapy, and stem cell therapy have shown good results in preclinical animal experiments, these treatments have not yet entered the clinical trial stage and are still some distance away from successful translation and application. Developing effective novel medications for AS management necessitates a thorough understanding of the exact pathogenetic processes involved. Pathways such as Transforming growth factor‐β (TGF‐β) and Wingless ‐ type MMTV integration site family (Wnt)/β‐catenin have been extensively studied in various fibrotic diseases, including LTS.^10^, ^11^, ^12^, ^13^, ^14^ A deeper understanding of pathogenetic signal transduction could potentially lead to the successful development of medications targeting specific functional molecules to modulate these pathways. For instance, plorofucofuroeckol A can inhibit Smad2/3 phosphorylation in fibroblasts, thereby regulating downstream signaling in the TGF‐β pathway and preventing scar formation.^15^, ^16^ Additionally, the dysregulation of immune responses and microbial communities offers insights for designing more comprehensive treatment modalities beyond solo antifibrotic therapies.

Currently, there is a lack of up‐to‐date and comprehensive literature reviews summarizing advancements in the mechanistic research and targeted therapy development for AS. To assist researchers in identifying gaps in both the etiology of AS development and therapeutic agent development, this literature review focuses primarily on classification, pathogenesis and management of AS. We first introduced the classification of AS from the perspectives of otolaryngologists, interventional pulmonologists, and thoracic surgeons. After that, we reviewed the latest progress in the pathogenesis of AS, including fibrosis, inflammation, epithelial–mesenchymal transition (EMT), metabolic reprogramming, microbiome, genetic susceptibility, and other mechanisms. Then, we sorted out the current preclinical research on AS, including antifibrotic therapy, anti‐inflammatory therapy, anti‐EMT therapy, antimetabolic therapy, stem cell therapy, and other preclinical treatments. Furthermore, we summarized the current clinical treatments, including endoscopic therapy, surgical therapy, and nonsurgical therapy. Finally, we looked forward to the prospects of exploring crosstalk between different cell types and between the microorganisms and cells through single‐cell RNA sequencing, 16S rRNA gene sequencing and shotgun metagenomic sequencing to identify the intersection of multiple mechanisms.

## 2 CLASSIFICATION OF THE AS

AS is a multidisciplinary condition that involves the fields of otolaryngology, thoracic surgery, and interventional pulmonology.^1^ The classification of AS is intricate due to the involvement of multiple organs and the heterogeneity of classification systems employed in various specialties. Despite the suggestion of several systems to quantify and characterize stenotic lesions, a universally acknowledged standard is still elusive. (Table 1 is inspired by Agrawal et al.^1^ ) Current investigations into AS classification are predominantly centered on the supraglottic, glottic, subglottic, trachea, and bronchial regions. Constrictions in these initial four sites are commonly labeled as LTS, a focal point for otolaryngologists’ classification endeavors. On the other hand, thoracic surgeons and pulmonologists primarily focus on tracheal and bronchial stenosis.

**TABLE 1:** Classification of airway stenosis.

	Author/year	Classification criteria
Otolaryngologists’ perspective	Myer et al. 1994^17^	The percentage of stenosis: Grade I: 0–50% Grade II: 51–70% Grade III: 71–99% Grade IV: 100%
	McCaffrey et al. 1992^18^	The percentage of stenosis: Grade I: 0–50% Grade II: 51–70% Grade III: 71–99% Grade IV: 100%
	McCaffrey et al. 1992^18^	The vertical extent of stenosis: Stage I: Lesions are confined to the subglottis or trachea and less than 1.0 cm Stage II: Lesions are isolated to the subglottis and longer than 1.0 cm Stage III: Subglottic or tracheal lesions not involving the glottis Stage IV: Lesions involve the glottis
	Lano et al. 1998^19^	The number of involved sites (glottis, subglottis, trachea): Stage I: One site Stage II: Two sites Stage III: Three sites
	Nouraei et al. 2007^20^	Documenting functional outcome (ADVS): Airway status (A) 1. No airway prosthesis 2. Intraluminal airway prosthesis (stent) 3. Tracheostomy or T‐tube dependent, patient voices 4. Tracheostomy‐dependent, patient does not voice 5. Death as a result of a direct complication of airway disease Dyspnea (D) 1. I get short of breath only on strenuous exercise 2. I get short of breath when hurrying on the level or up a slight hill 3. I walk slower than people of the same age on the level because of breathlessness, or have to stop for breath when walking at my own pace on the level 4. I stop for breath after walking 100 yards or after a few minutes on the level 5. I am too breathless to leave the house Voice (V) 1. I have had no problems with my voice 2. I have had some problems with my voice. 3. I have quite a rough voice. I find making voice effortful and have significant difficulties being heard/understood in loud environments 4. I can only produce a weak voice/whisper despite my best efforts, and have difficulty being heard/understood in normal conversation or on the telephone 5. I have no voice Swallowing (S) 1. I have been able to eat and drink normally 2. I have been able to eat a normal diet but with some difficulty. 3. I have had significant swallowing difficulties. 4. My swallowing is a serious problem/is seriously abnormal. 5. I am unable to swallow.
	Monnier et al. 2015^21^	Myer‐Cotton system: Grade I: 0–50% Grade II: 51–70% Grade III: 71–99% Grade IV: 100% The number of involved sites, including supraglottis, glottis, subglottis and trachea (similar to Lano system) a: One site b: Two sites c: Three sites d: All four sites +: The presence of severe comorbidities, or congenital abnormalities
Thoracic surgeons’ and pulmonologists’ perspective	Freitag et al. 2007^22^	Types of stenosis: Structural Type 1: Exophytic/intraluminal Type 2: Extrinsic Type 3: Distortion Type 4: Scar/stricture Dynamic or functional Type 1: Damaged cartilage/malacia Type 2: Floppy membrane Degree of stenosis: Code 0: 0% Code 1: 0–25% Code 2: 26–50% Code 3: 51–75% Code 4: 76–90% Code 5: 90–100% Location: I: Upper third of the trachea II: Middle third of the trachea III: Lower third of the trachea IV: Right main bronchus V: Left main bronchus
	Ghorbani et al. 2012^23^	Diameter of stricture: Score 0: 0–25% Score 1: 26–50% Score 2: 51–75% Score 3: 76–90% Score 4: 91–100% Type of stenosis: Score 1: Granulation tissue Score 2: Granulation tissue, fibrosis and inflammation Score 3: Fibrosis Score 4: Malacia Clinical symptoms: Score 1: Dyspnea only during intense activity Score 2: Dyspnea during normal activity but physical examination was normal Score 3: Long inhalation and exhalation but with no stridor or retraction Score 4: Presence of stridor and retraction

### 2.1 Classification from the perspective of otolaryngologists

The widely used classification systems are the Myer‐Cotton system and the McCafrey system. The former classifies AS into four grades based on the percentage of stenosis: grade I to 50%; grade II from 51 to 70%; grade III from 71 to 99%; grade IV for total obstruction,^17^ while the latter divides it into four stages based on the vertical extent of stenosis: stage I: lesions are confined to the subglottis or trachea and less than 1.0 cm; stage II: lesions are isolated to the subglottis and longer than 1.0 cm; stage III: subglottic or tracheal lesions not involving the glottis; stage IV: lesions involve the glottis.^18^ Both classification systems are based on accurate measurements, but in practice these data are not always available clinically, so the Lano system, which does not require precise measurements and is easy to use and remember, was developed to divide AS into three stages based on the number of involved sites (glottis, subglottis, trachea). Stage I: one site; stage II: two sites; stage III: three sites.^19^ One study evaluated these three systems and found that the McCaffrey and Lano systems were more accurate than the Cotton‐Myer system.^4^ In addition, another classification system classifies from the perspective of documenting functional outcome adult LTS, including four domains of airway status, dyspnea, voice, and swallowing.^20^ A consensus paper of the European Laryngological Society integrated the Myer‐cotton system and the Lano system, and optimized the Lano system by adding the stenosis involved sites to supraglottis, glottis, subglottis, and trachea, and adding the evaluation criteria for whether patients have other serious comorbidities or congenital abnormalities.^21^ Finally, a standardized airway classification system was constructed.

### 2.2 Classification from the perspectives of thoracic surgeons and pulmonologists

Both the Freitag system and the Ghorbani system's classification criteria for AS include the degree of stenosis, that is, the percentage of obstruction, which is similar to the Myer‐Cotton system. From the perspective of etiology, Freitag system classifies the stenosis types into structural and dynamic categories, and further subdivides the trachea and bronchus into five specific sites.^22^ In contrast, the Ghorbani system pays special attention to tracheal stenosis after intubation, categorizing stenosis types based on the type of lesion tissue and clinical symptoms.^23^

Although the current classification of AS focuses on supraglottic, glottic, subglottic, tracheal, and bronchial stenosis, more specifically LTS, anatomically speaking, the airway is composed of the upper airway and the lower airway, the former includes the nasal cavity, oral cavity, pharynx, and larynx, while the latter is composed of the trachea and bronchi. The current classification covers only the larynx, trachea, and bronchi, while both the nasal cavity and the pharynx have the possibility of stenosis, and there are almost no reports of narrowing in the oral cavity.

#### 2.2.1 Nasal cavity stenosis

Nasal stenosis can be either congenital or acquired. Congenital nasal stenosis usually results from abnormal bone structure, mainly caused by abnormal nasal development during embryonic development. The main types of stenosis include: choanal atresia and stenosis, congenital nasal pyriform aperture stenosis, congenital midnasal stenosis, arhinia, and nasal septum deviation.^24^ Acquired nasal stenosis may be caused by infection, trauma, or iatrogenic factors, with iatrogenic factors accounting for a larger proportion, including surgical procedures, nasal intubation, or radiation therapy.^25^

#### 2.2.2 Pharyngeal stenosis

Pharyngeal stenosis can be divided into nasopharyngeal stenosis, oropharyngeal stenosis, and hypopharynx stenosis. Nasopharyngeal stenosis is caused by the fusion of the tonsils and soft palate with the posterior pharyngeal wall, resulting in the obstruction of the normal passage between the nasopharynx and oropharynx.^26^ Oropharynx stenosis is caused by adhesion of the anterior pillars and inferior tonsillar fossa to the base of the tongue, which accordingly narrows the oropharyngeal aperture.^27^ These two types of strictures are almost always caused by iatrogenic injuries, such as surgery or radiation therapy, including tonsillectomy, adenoidectomy, uvulopalatopharyngoplasty, pharyngeal reconstruction for velopharyngeal insufficiency, or other procedures in the pharynx, as well as radiation therapy for nasopharyngeal carcinoma.^28^ Hypopharyngeal stenosis is also caused by surgery and radiation therapy and is commonly seen in hypopharyngeal squamous cell carcinoma, usually after total laryngectomy or chemoradiotherapy.^29^, ^30^, ^31^

## 3 PATHOGENESIS OF AS

Currently, research on the pathogenesis of AS primarily focuses on LTS, and due to the commonalities in the pathogenesis of AS, we primarily introduce the existing related mechanism research on LTS. The main mechanisms include fibrosis, inflammation, EMT, metabolic reprogramming, microbiome, and genetics.

### 3.1 Fibrosis

As one of the LTS phenotypes, fibrosis results from the excessive accumulation of extracellular matrix (ECM) components such as collagen and fibronectin.^32^ This process represents a normal and crucial phase of tissue repair across all organs, but excessive activation during the disease process leads to aberrant ECM deposition, resulting in abnormal narrowing of passageway.^5^, ^32^ Fibroblasts are activated as effector cells, differentiating into myofibroblasts, which are characterized by the expression of contractile proteins and the massive release of ECM proteins.^33^ In the occurrence of LTS, myofibroblasts that should have undergone apoptosis persist and lead to pathological tissue remodeling.^5^ Numerous studies have explored fibrosis mechanism in LTS, trying to elaborate fibrosis‐related mechanisms and signaling pathways, and to develop targeted drugs. We mainly focused on TGF‐β signaling pathway and Wnt signaling pathway, which has been extensively studied.

#### 3.1.1 TGF‐β signaling pathway

The significance of TGF‐β signaling pathway in pathogenesis of LTS is indicated by the high expression of TGF‐β1 in biopsy specimens from patients with subglottic and tracheal stenosis and stent‐related stenoses compared with control sections.^10^ Based on sequence similarity and activated signaling pathways, the TGF‐β family comprises two subfamilies, one of which is the TGF‐β/Activin/Nodal subfamily while another is the BMP (bone morphogenetic protein)/GDF (growth and differentiation factor)/MIS (Muellerian inhibiting substance) subfamily.^34^ The TGF‐β signaling pathway can be divided into canonical and noncanonical pathway according to whether there are participating Smads, consisting of three categories: the receptor‐regulated Smad (R‐Smad), the Co‐mediator Smad (Co‐Smad), and the inhibitory Smad (I‐Smad).

In the canonical pathway, TGF‐β subfamily binds to TβRII of functional complex of TGF‐β family receptors, which consists of two type II and two type I transmembrane serine/threonine kinase receptors, TβRII and TβRI.^35^ Activated TβRII recruits and phosphorylates TβRI, allowing these two types of receptors to form a heteromeric complex.^36^ With the help of the heteromeric complex, downstream effector R‐Smads, especially for Smad2/Smad3, are phosphorylated and formed into homotrimerization with the Co‐Smad, Smad4. After translocated into the nucleus, the trimeric complexes recruit cofactors, such as the histone acetyl transferases (HATs) p300 and CREB‐binding protein (CBP) to regulate the expression of fibrosis‐related genes.^34^, ^37^ The remaining R‐Smads, Smad1, Smad5, and Smad8, are activated by the BMP subfamily and, in cooperation with Smad4, translocate into the nucleus to regulate the expression of specific genes.^38^ The I‐Smads, Smad6 and Smad7, inhibit the TGF‐β signaling via various mechanisms, including interfering with interactions between R‐Smads and TβRI, downregulation of TβRI, prevention of Smad trimeric complexes and transcriptional regulation in the nucleus.^39^

In the noncanonical pathway, in addition to Smad‐mediated signaling, TGF‐β ligands can also activate other signaling pathways, including phosphoinositide‐3‐kinase (PI3K)/Akt pathway, MAPK pathways (ERK1/2, JNK and p38/MAPK signaling pathways), Rho‐like signaling pathway.^40^ Here, we mainly discuss the TGF‐β‐induced PI3K/Akt/mammalian target of rapamycin (mTOR) signaling pathway, which has been extensively studied on the targets for pharmacologic intervention of LTS.^41^, ^42^ TGF‐β can activate Akt, also known as the serine/threonine kinase protein kinase B through The PI3K. Subsequently, activated Akt exerts its effects of fibrosis and promoting cell proliferation through downstream mTOR, whose complex mTORC1 regulates phosphorylation of S6 kinase (S6K) and eukaryotic initiation factor 4E‐binding protein 1 (4E‐BP1) to affect protein synthesis.^40^, ^43^, ^44^ Moreover, recent study has shown the role of TGF‐β/RhoA pathway in LTS.^45^

Before conducting the signal transduction described above, TGF‐β must be activated from a latent complex, which is composed of TGF‐β homodimer, latency‐associated peptide (LAP), latent TGF‐β binding protein (LTBP) and stored in the ECM. Latent TGF‐β transformed into bioactive homodimeric ligands, binding to the TβR complex with the help of proteins and enzymes (thrombospondin 1 [TSP1], glycoprotein A repetitions predominant protein (GARP), integrins, and other TGF‐β‐binding proteins).^36^Among them, the most thoroughly studied mechanism for activation of TGF‐β1 is the interaction between the αv‐containing subset of integrins and the TGF‐β latent complex. Specifically, the integrins αvβ1, αvβ3, αvβ5, αvβ6, and αvβ8 can bind to LAP and αvβ6 can release active TGF‐β1 under the action of mechanical force generated by αvβ6 integrin‐expressing cells, while it is not applicable to αvβ8.^32^

**FIGURE 1:**
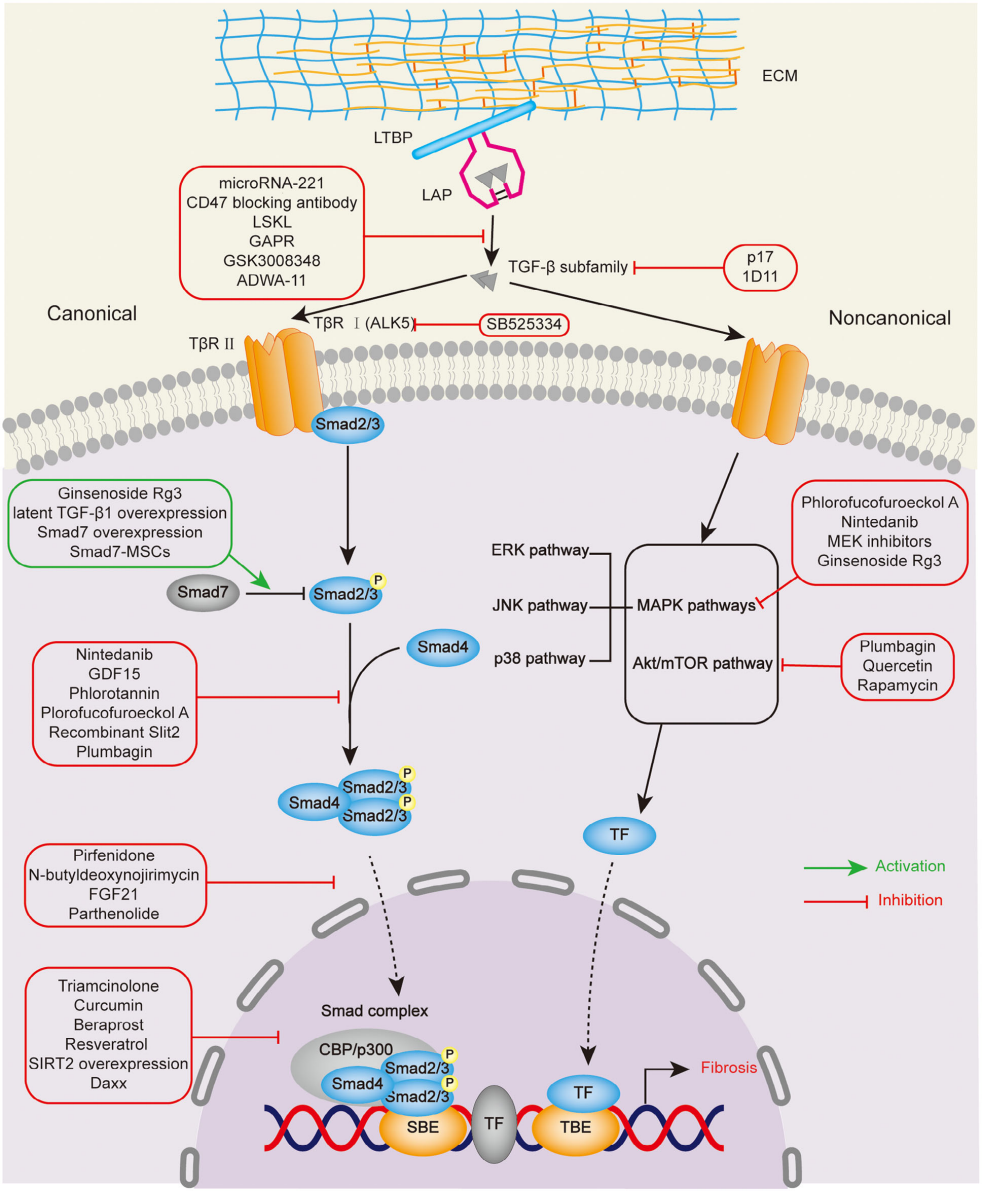
Schematic representation of canonical and noncanoical TGF‐β signaling pathway and targeted therapy in AS (BMP signaling pathway is not shown). The TGF‐β ligands are synthesized as a latent complex consisting of TGF‐β homodimer, LAP and LTBP, which are stored in ECM. In the canonical pathway, active TGF‐β dimers induce the formation of heteromeric complex by sequentially activating TβII and TβI, the heteromeric complex then reacts with the downstream Smad proteins, which form into trimeric complexes, translocate into nucleus and associate with the SBE, ultimately regulating the transcription of fibrosis‐related genes. The therapeutic mechanisms targeting this process can be divided into five categories: (1) inhibition of TGF‐β and TβR, (2) inhibition of Smad2/3 phosphorylation, (3) inhibition of nuclear translocation, (4) inhibition of the transcriptional activity of Smad complexes, and (5) enhancement of I‐Smad expression. In the noncanonical pathway, TGF‐β ligands can transmit a signal through other signaling pathway, including MAPK pathways and PI3K/Akt pathway. Targeting these signaling pathways can become a therapeutic strategy.

TGF‐β plays crucial roles in multiple organs and tissue types, mediating fibroblasts differentiation into myofibroblasts, which express high level of growth cytokines and synthesize excessive collagen, resulting in ECM deposition. With the discovery of high expression of TGF‐β in pathological tissues and the efficacy of drugs that inhibit the TGF‐β signaling pathway in animal model,^5^ it suggests that the role of TGF‐β in the pathogenesis of LTS is crucial, and targeting components in the TGF‐β signaling pathway might have potential therapeutic effects (Figure 1).

#### 3.1.2 Wnt/β‐catenin signaling pathway

**FIGURE 2:**
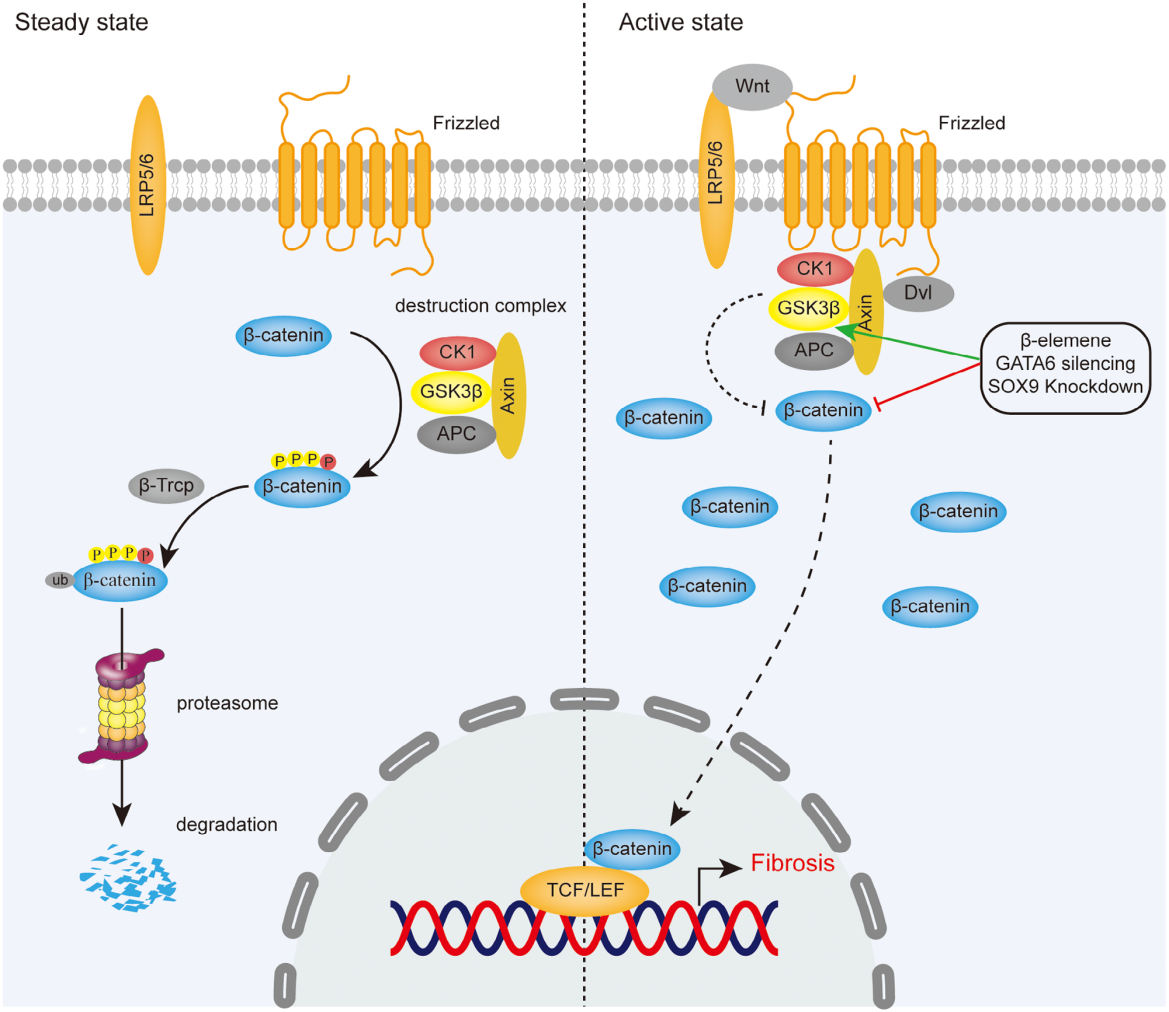
Overview of canonical Wnt signaling pathway and targeted therapy in AS. In steady state, absence of Wnt ligands causes β‐catenin to be phosphorylated by the destruction complex, ubiquitinated by β‐Trcp, and finally degraded by the proteasome. While in active state, Wnt proteins induce a spatial interaction between the receptors Frizzled proteins and LRP5/6, which hinders the degradation of β‐catenin by destruction complex, allowing accumulated β‐catenin to translocate into the nucleus and interact with transcription factors TCF/LEF, upregulating the transcription of fibrosis‐related genes. Through enhancing activity of GSK3 β and directly inhibiting β‐catenin as a targeted therapeutic strategy.

The Wnt signaling pathway is essential in embryonic development and adult tissue homeostasis,^46^ which regulates airway epithelial differentiation, cartilage formation and normal upper airway development in laryngotracheal region.^11^, ^12^, ^13^, ^14^ According to β‐catenin involvement, Wnt signaling pathways can be divided into canonical and noncanonical pathways, the former, also known as Wnt/β‐catenin signaling pathway, mainly controls cell proliferation, while the latter, including Wnt/Ca^2+^ pathway and noncanonical Wnt planar cell polarity, regulates cell polarity and migration, forming a network of mutual regulation between them.^46^ Increasing evidence has shown that Wnt/β‐catenin signaling pathway plays a critical role in fibrosis of LTS.^47^, ^48^ Given the relatively extensive research on canonical pathway, it will be mainly discussed in Wnt/β‐catenin signaling pathway causing fibrosis and its targeted therapy (Figure 2).

The key events of the canonical pathway are the accumulation and translocation of transcription factor β‐catenin into the nucleus, which induce activation of T cell factor (TCF)‐lymphoid enhancer factor (LEF)‐dependent gene expression, leading to fibrosis caused by proliferation and activation of fibroblasts in trachea.^47^, ^49^ In steady state, β‐catenin is phosphorylated and ubiquitinated by the “destruction complex” and the E3–ubiquitin ligase b‐TrCP, subsequently degraded in proteasomes. The destruction complex comprises the proteins glycogen synthase kinase 3β (GSK3β), adenomatous polyposis coli (APC), casein kinase 1, and axin.^49^ While in active state, Wnt proteins, as secreted glycoproteins and growth stimulatory factors, induce a spatial interaction between the receptors Frizzled proteins (FZD) and lipo‐protein receptor‐related protein 5/6 (LRP5/6), recruiting destruction complex to cell membrane by interacting with FZD and resulting in loss of the ability to degrade β‐catenin.^46^, ^49^

#### 3.1.3 ECM dysregulation

ECM is a dynamic three‐dimensional structure that continually remodels to maintain tissue homeostasis, offering physical support for tissue integrity and resilience. Its components continuously interact with epithelial cells by serving as ligands for cell receptors, such as integrins, thereby transmitting signals that regulate adhesion, migration, proliferation, apoptosis, survival, or differentiation.^50^

##### For the relationship between the components of ECM and fibrosis

The main components of ECM include fibrillar proteins, glycoproteins, matricellular proteins, cross‐linking enzymes, and proteolytic cleavage enzymes.^51^ Fibulin belongs to the category of glycoproteins. It has been reported that the deletion of the Fbln1c and the therapeutic inhibition of Fbln1c can reduce airway collagen deposition in mice allergic airway disease model.^52^ In matricellular proteins, cellular communication network factor 2 (CCN2) is highly expressed in the subglottis of patients with SGS, and inhibiting CCN2 can alleviate the condition^53^; Periostin (POSTN) is also a type of matricellular protein. A recent study indicate that POSTN is a crucial molecule in the formation of LTS scars, which mediates the formation of scar‐induced LTS by regulating the TGF‐β/RHOA pathway through its interaction with TGF‐β1.^45^ As for proteolytic cleavage enzymes, matrix metalloproteinases (MMPs) are the main enzymes involved in ECM degradation. Tissue metalloproteinase inhibitors (TIMPs) can inhibit MMPs, resulting in ECM accumulation. The imbalance between MMPs and TIMPs is one of the main mechanisms of ECM abnormal remodeling. When human primary tracheal epithelial cells are exposed to bile acids, the level of MMP9 increases, and the inhibition of MMP9 can reduce ECM remodeling.^53^, ^54^

##### For the relationship between ECM mechanical properties and fibrosis

In fibrosis, changes in gene and protein expression are expected to cause ECM stiffness, creating a cellular microenvironment capable of exerting considerable mechanical forces on resident cells.^55^ Integrin‐mediated mechanotransduction plays a crucial role in fibrosis, enabling the activation of latent TGF‐β and generating the biological activity associated with fibrosis.^55^ Furthermore, mechanobiological research on pulmonary fibrosis has pinpointed several crucial signaling pathways, namely, Rho/ROCK, MRTF‐A, and YAP/TAZ.^56^ Consequently, targeting ECM stiffness and mechanotransduction signaling pathways emerges as a potential therapeutic approach for AS. Research on the mechanism of ECM in AS is scarce, further understanding of the mechanisms of ECM dysregulation in AS and in‐depth research on related targeted drugs will aid in the development of new therapeutic targets for AS in clinical practice.

### 3.2 Inflammation

In recent years, there has been increasing attention paid to the importance of inflammatory phenotype in the development of AS. The formation of scar tissue may often be related to inflammation, mainly affecting its immune microenvironment, which consists of various immune cells, immune cytokines and ECM. After the airway is subjected to various injuries, especially tracheostomy and intubation, it can cause tracheal tissue necrosis, leading to its activation by various damage‐associated molecular patterns (DAMP) and pathogen‐associated molecular patterns (PAMP), which interact with Toll‐like receptors (TLR) to activate the NF‐κB signaling pathway, leading to the activation of inflammasome, as well as the release of IL‐1β, IL‐18, and various other proinflammatory cytokines and chemokines, and further promoting the activation of immune cells and their immune cytokines in adaptive and innate immunity, thereby promoting the formation of fibrosis. In recent years, the effects of certain immune cells and inflammatory factors on fibrosis have been identified in LTS. It was observed that several markers of inflammation were upregulated in specimen from LTS compared with normal biopsy samples, such as IL‐1, IL‐4, INF‐γ, and CCL2.^57^, ^58^, ^59^ The high expression of Th1 cytokine INF‐γ and Th2 cytokine IL‐4 suggests that adaptive immune response may play an important role in LTS, which was verified in severe combined immunodeficiency mice.^60^ In addition, innate immune response represented by macrophage polarization has also been demonstrated to play a role in LTS.^61^ Furthermore, the transcriptomic profiling of SGS patients reveals a distinctively proinflammatory gene signature and hyper‐activation of NF‐κB with its downstream inflammasome.^62^

#### 3.2.1 NF‐κB signaling pathway and inflammasome

**FIGURE 3:**
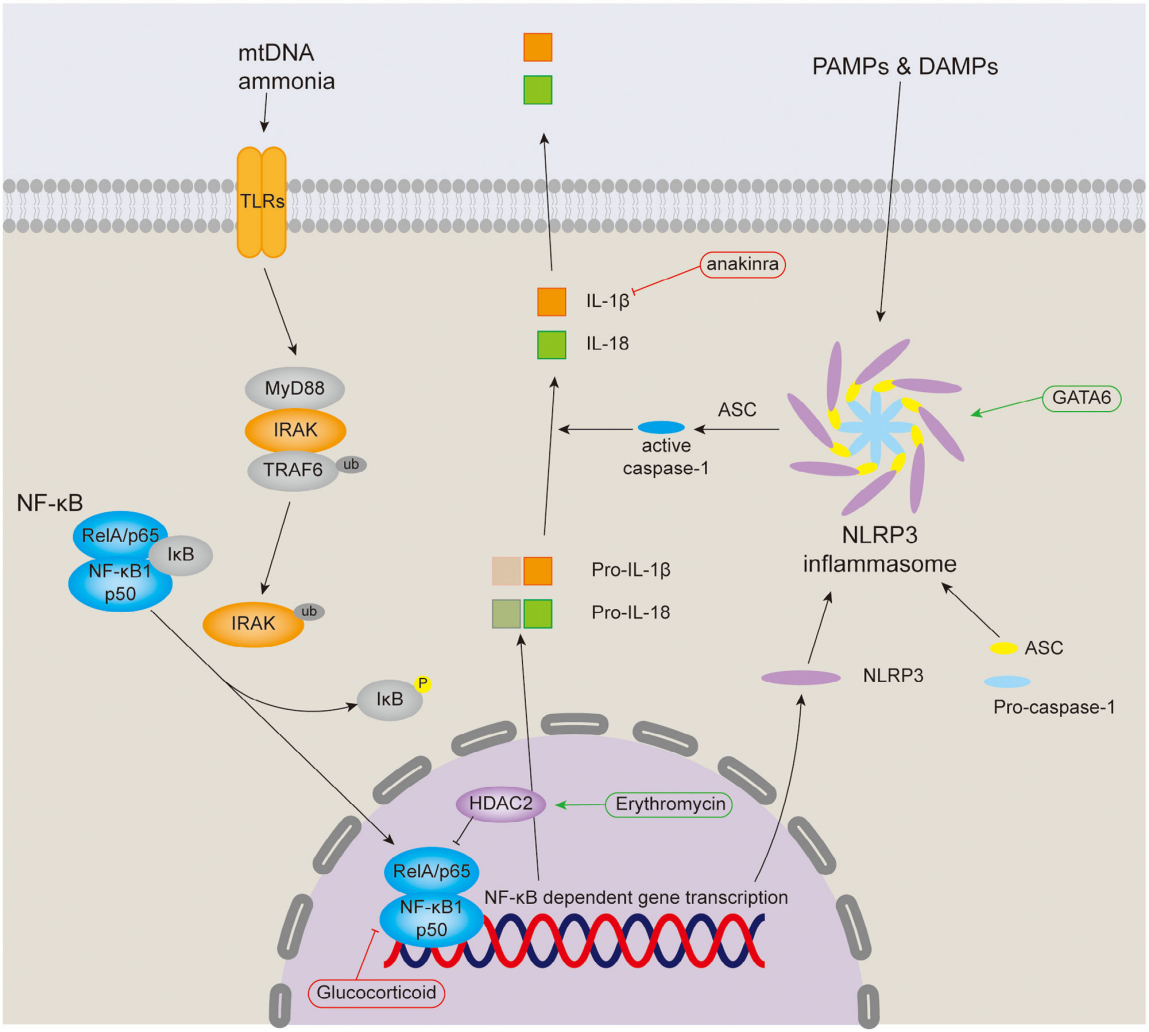
NF‐κB signaling pathway and NLRP3 inflammasome in AS. The activation of inflammasome requires two signals. The first signal is that ammonia and mtDNA bind to TLRs, thereby activating NF‐κB signaling and promoting the transcrition of NLRP3, pro‐IL‐1β, and pro‐IL‐18. Another signal is that NLRP3 is directly activated by PAMPs and DAMPs, and then converts procaspase‐1 recruited by ASC into caspase‐1, which catalyzes the conversion of pro‐IL‐1 β and pro‐IL‐18 into their mature products IL‐1 β and IL‐18.

Biological, physical, and chemical factors can activate the NF‐κB signaling pathway when causing tracheal injury,^63^, ^64^, ^65^ which increases the incidence of tracheal stenosis. The NF‐κB signaling pathway can be divided into canonical and noncanonical pathways, and we will mainly introduce the canonical pathway. The NF‐κB superfamily comprises five transcription factors: NF‐κB1 (p50), NF‐κB2 (p52), RelA (p65), RelB and REL (c‐Rel), RelA, and p50 heterodimers are responsible for the transcription of target genes in the canonical NF‐κB pathway, while RelB and p52 form a heterodimer in the noncanonical NF‐κB pathway.^66^ The canonical NF‐κB pathway is stimulated by diverse factors, such as pattern recognition receptors (like TLR), ligands of various cytokine receptors, TNF receptor superfamily, as well as T‐cell receptor and B‐cell receptor, and the key event is the IκB kinase (IKK)‐mediated phosphorylation of IκB protein, which sequesters RelA and p50 in the cytoplasm.^67^ In tracheal injury, ammonia and local release of mtDNA activate NF‐κB pathway through TLR.^63^, ^64^ MyD88, one of the TLR adapters, activates IRAK family of kinases, which in turn stimulate the E3 ubiquitin ligase activity of TRAF6, allowing TRAF6 to undergo self‐ubiquitination and activate a ubiquitin‐dependent kinase, TAK1.^68^ TAK1 activates the downstream kinase IKK, causing the key event mentioned above to occur and promoting the transcription of NF‐κB‐dependent genes, such as NLRP3, pro‐IL‐1β, and pro‐IL‐18, which are required for inflammasome activation (Figure 3).^68^

Inflammasomes are a group of intracellular multiprotein complexes that assemble in response to PAMPs and DAMPs, and their activation leads to pyroptosis in tracheal injury.^65^, ^68^, ^69^ Canonical inflammasomes comprises the inflammasome sensor receptor, the adapter protein ASC (apoptosis‐associated speck‐like protein containing a CARD) and procaspase‐1, upon stimulation of the inflammasome receptor NLRP3, which has been demonstrated in tracheal tissue, oligomerizes and recruits procaspase‐1 through ASC, thereby stimulating procaspase‐1 processing and conversion to active caspase‐1.^65^, ^66^, ^69^ Activated caspase‐1 then cleaves pro‐IL‐1β and pro‐IL‐18, leading to the secretion of active IL‐1β and IL‐18, of which IL‐1β was observed to be upregulated in the serum of LTS patients.^59^, ^66^

#### 3.2.2 Dysregulation of the immune microenvironment

DAMPs and PAMPs activate NF‐κB and inflammasome, which can release various proinflammatory factors and chemokines. Immune cells and their immune cytokines in adaptive and innate immunity can also be activated. These immune cells can be recruited to the wound site, forming an immune microenvironment with immune cytokines and other cytokines, thereby further promoting fibrosis. In recent years, with the help of bulk tissue RNA sequencing, single‐cell RNA sequencing combined with bioinformatics and flow cytometry, the key roles of innate and adaptive immune responses can be determined in LTS, and key immune cells and their subtypes as well as cytokines.^70^, ^71^, ^72^

For immune cells and immune factors involved in adaptive immunity within the immune microenvironment, CD8^+^ tissue resident memory T cells were demonstrated to be significantly enriched in human subglottis in idiopathic subglottic stenosis (iSGS).^71^ For CD4^+^ T cells, a study has shown a local imbalance of Th1/Th2 cells in TS,^73^ Th2 cells and the cytokine IL‐4 they produce are associated with iatrogenic LTS, while Th1 cells and the cytokine INF‐γ they produce can attenuate LTS by reducing the proliferation of derived fibroblasts, the production of soluble collagen and collagen protein expression as well as suppressing the expression of the profibrotic cytokine TGF‐β.^74^, ^75^ Given that INF‐γ is unlikely to be used in systemic therapy due to its severe side effects, it is more reasonable to modulate the Th1/Th2 imbalance and increase Th1 responses. In pulmonary fibrosis, intranasal vaccinia vaccination has been shown to induce Th1 responses and suppress M2 macrophage responses in the lungs and thereby mitigating disease.^76^ Produced by Th17 cells and γδ T cells, IL‐17A promotes iSGS and LTS through directly driving scar fibroblast proliferation or significant activating the canonical IL‐23/IL‐17A pathway, whereas sirolimus targeting mTOR may effectively treat LTS by inhibiting profibrotic Th17 cells.^77^, ^78^, ^79^

For immune cells and immune factors involved in innate immunity within the immune microenvironment, the role of macrophages in LTS is gradually being noticed. CD4^+^ T cells can activate and recruit macrophages, the Th1 cytokine INF‐γ activates nitric oxide synthase 2 expression in “classically activated” macrophages (M1), and the Th 2 cytokine IL‐4 preferentially stimulate arginase 1 (ARG1) activity in “alternatively activated” macrophages (M2), the ARG1 of M2 macrophages promotes the production of l‐ornithine, which is beneficial to the production of polyamines and l‐proline. Polyamines are essential for cell growth, and proline is a substrate for collagen synthesis, which makes M2 macrophages are important in repair processes.^80^ Studies have shown that macrophages are upregulated in LTS specimens, and dysregulated M2 macrophages not only play a role in abnormal laryngotracheal wound healing, but also promote collagen expression in airway fibroblasts, thereby playing a key role in LTS.^61^, ^81^, ^82^ A previous study showed the paracrine signaling interaction between macrophages and fibroblasts derived from vocal fold scar, and fibroblasts can interfere with macrophage activation and phenotype.^83^ A recent study identifies a new subpopulation of S100A8/9 expressing macrophages, which secret protein S100A8/9 and increase collagen gene expression in iSGS‐derived fibroblasts thereby eliciting profibrotic effects.^72^ Taken together, targeting macrophages may be a therapeutic strategy to alleviate fibrosis in LTS.

The role of other cytokines in LTS has also been gradually discovered, IL‐6 may mediate transdifferentiation of normal laryngotracheal fibroblasts into a myofibroblast phenotype under hypoxic conditions in vitro.^84^ After being found to be elevated in the serum and granulation tissue of TS patients, IL‐11 can promote EMT and the transformation of fibroblasts into myofibroblasts, and the inhibition of IL‐11‐mediated signaling pathways can improve TS in rats.^85^ And a study showed that activation of the chemokine receptor CXCR7 inhibits E‐cadherin expression through the NF‐κB signaling pathway, thereby promoting the migration of LTS‐derived fibroblasts.^86^ Furthermore, studies have shown that the expression of PD‐1 and PD‐L1 is increased in patients with LTS, suggesting that checkpoint inhibitors targeting the PD‐1/PD‐L1 axis may play a role in the treatment of LTS.^87^, ^88^

### 3.3 Epithelial–mesenchymal transition

EMT is the process of transdifferentiation of epithelial cells into motile mesenchymal cells, which physiologically promotes development, wound healing, and stem cell behavior, and pathologically promotes fibrosis and cancer progression.^89^ According to these effects, it can be divided into three different subtypes. Type 1 EMT is associated with implantation, embryo formation, and organ development and can generate mesenchymal cells, type 2 EMT is associated with tissue regeneration, fibrosis and inflammation and can produce myofibroblasts, and its mechanism is closely related to LTS, type 3 EMT is associated with cancer progression and metastasis.^90^ Key events of EMT include deconstruction of cell junctions and polarity, changes and motility of the cytoskeleton, downregulation of epithelial markers and upregulation of mesenchymal markers.^89^ Changes in some common biomarkers and transcription factors are involved in the EMT process. Among epithelial markers, as the prototypical epithelial cell marker of EMT, E‐cadherin expression decreased, while N‐cadherin expression increased, achieving the transformation of E‐cadherin to N‐cadherin, and reduced expression of tight junction proteins occludin and claudins; Among the mesenchymal markers, vimentin, α‐SMA and S100A4, also known as FSP1, were found to have increased expression, while expression of cytokeratin decreased. The extracellular protein fibronectin and MMP9 were found to have increased expression. Transcription factors including SNAIL, TWIST, and zinc‐finger E‐box‐binding (ZEB) are activated early in EMT, thereby contributing to gene expression changes that suppress epithelial phenotypes and activate mesenchymal phenotypes.^89^, ^90^

Given that gastroesophageal reflux is a risk factor for LTS, studies on the components of gastric refluxate have shown that bile acids can induce EMT in human primary airway epithelial cells, which may be the basis for the development of LTS,^54^ while pepsin has not been found to affect EMT and fibrosis.^91^ However, another study showed that pepsin mediates laryngeal epithelial disruption and E‐cadherin cleavage, which is inhibited by amprenavir.^92^ And epithelial barrier dysfunction has been found in LTS.^93^, ^94^ Taken together, EMT has an important role in LTS.

Biomarkers of EMT also detected in LTS. The expression of epithelial markers E‐cadherin, claudin, occludin, and epithelial cell adhesion molecule were downregulated, and the expression of mesenchymal markers vimentin, α‐SMA, and S100A4 were upregulated, and the expression levels of transcription factors ZEB1 and Snail1 were also increased.^94^, ^95^, ^96^, ^97^ In addition, as a novel transcription factor regulating EMT, tracheal epithelial GATA6 triggers EMT through NLRP3 activation.^69^

Signaling pathways inducing EMT in LTS. The EMT process involves TGF‐β, NF‐κB, and β‐catenin signaling pathways. Targeting these pathways can inhibit EMT and improve LTS, which has therapeutic significance.

### 3.4 Metabolic reprogramming

Metabolic reprogramming is a hallmark found in malignancy, whereby stereotyped metabolic activities in cancer cells, specifically aerobic glycolysis, glutamine catabolism, macromolecule synthesis, and redox homeostasis, support the requirements for exponential growth and proliferation.^98^ The discovery of aerobic glycolysis and glutamine catabolism in LTS‐derived fibroblasts demonstrates the role of metabolic reprogramming in LTS.^99^, ^100^, ^101^, ^102^

Metabolic alterations of fibroblasts. It has been reported that LTS‐derived fibroblasts have reduced oxidative phosphorylation and increased glycolysis/oxidative phosphorylation ratio compared with normal fibroblasts, and fibroblasts drive their proliferation through aerobic glycolysis, similar to Warburg's effect.^99^ However, iLTS scar fibroblasts from patients with type 2 diabetes mellitus (T2DM) preferentially utilize oxidative phosphorylation, exhibit greater contractility, and have a unique myofibroblast phenotype compared with controls.^103^

Metabolome profiling of differentially expressed substances. With the help of metabolomic analysis, it was found that carnitine levels in tracheal scar tissue was significantly lower than those in scar‐free tissue, while the pathway enrichment analysis of carnitine revealed a significant enrichment in fatty acid oxidation.^104^ And carnitine treatment has been shown to reduce the severity of LTS caused by tracheostomy or trauma.^105^, ^106^ Additionally, T2DM iLTS‐scar fibroblasts were identified to have increased itaconate, a metabolite associated with immune‐induced scar remodeling and can be identified by elevated CD90 (Thy‐1) expression in vitro.^107^

### 3.5 The microbiome

The presence of bacterial biofilms and increased bacterial counts in patients with tracheal stenosis suggests a correlation between tracheal stenosis and microbial communities.^108^ Advanced technologies for identifying microbial sequences, including 16S rRNA gene and shotgun metagenomic analysis, have been applied in LTS to identify many microbial organisms that traditional microbial cultures cannot detect.

Microbial communities identified in LTS. EDIN‐producing MRSA were isolated from purulent tracheal secretions through microbial cultures in infants with acquired subglottic stenosis caused by intubation.^109^ Another study has demonstrated a unique correlation between iSGS and Mycobacterium species, which is a variant member of the Mycobacterium tuberculosis complex or a closely related novel Mycobacterium.^110^ 16S rRNA gene sequencing analysis of 61 swab samples from 17 iatrogenic and 10 adult idiopathic stenosis patients revealed that specific microbiota and community shifts are present with LTS in adults, with members of the family Moraxellaceae, including the known pathogens Moraxella and Acinetobacter, identified in idiopathic scar.^111^ Metagenomic shotgun sequencing of biofilms formed on pediatric tracheostomy tubes (TTs) revealed that in TTs with granuloma, Fusobacterium nucleatum, Haemophilus influenzae, Moraxella catarrhalis, and Streptococcus pneumoniae were predominant, most of which are considered pathogens.^112^ Metagenomic sequencing analysis was conducted on 16 protected specimen brush and eight broncho‐alveolar lavage samples from eight iSGS patients, and the results showed that the top four bacterial species were Neisseria subflava, Streptococcus oralis, Capnocytophaga gingivals, and Haemophilus aegyptius.^104^

Microbiology related to stent intubation for preventing stenosis. Preventive use of antibiotics, especially those controlling anaerobic and fungal organisms, can reduce the incidence of local infections and subsequent formation of granulation tissue in laryngotracheal reconstruction (LTR) using stents.^113^ The high rate of pathologic bacterial infiltration into postintubation laryngeal wounds suggests that the reduction of tracheal intubation bacterial colonization and early antibiotic treatment may improve acute laryngeal injury.^114^

### 3.6 Genetics

The significant homogeneity between patients with iSGS and the abnormal wound healing response partly depend on the genetic background of the affected individuals revealed that LTS may be associated with genetic factors.^115^, ^116^ A study showed that the rate of familial clustering of iSGS was quantified at 2.5%, and the inheritance was non‐Mendelian, and there might be genetic factors.^117^ And another study identified and provided the biologic context for 20 genes associated with proximal airway fibrotic diseases and laid the foundation for future detailed genetic studies.^118^

Several studies have attempted to identify single nucleotide polymorphisms (SNPs) associated with the occurrence of LTS, which identified functional SNPs of wound healing‐related genes, MMP1, MMP3, MMP12, CD14, TGF‐β1, and MCP1. A study found that a functional SNP of TGF‐β1, ‐509 C/T rs1800469 was associated with benign AS (BAS), in which the heterozygous C/T genotype has a protective function against BAS, while the C/C genotype may be a susceptibility factor for BAS.^119^ Another study showed that the MMP1 SNP rs1799750 was associated with the development of acquired LTS (ALTS), while the G/A genotype of the CD14 SNP rs2569190 may have a protective effect against ALTS.^120^ However, the last study did not find a significant association between candidate SNPs and the development of ALTS, and there are differences in susceptibility to ALTS among different ethnic backgrounds.^121^

### 3.7 Other mechanisms

Epigenetic modifications, hypoxia, autophagy, and cell cycle can also serve as mechanisms of AS, and drugs developed targeting these mechanisms have shown potential efficacy.

#### 3.7.1 Epigenetic modifications

Epigenetic alterations, such as DNA methylation and miRNAs, may contribute to the stabilization of the activated phenotype of fibroblasts in fibrotic diseases.^33^ DNA methylation analysis of samples from 12 patients with iSGS and four control patients revealed that there were five hypermethylated genes in iSGS biopsies compared with controls, including the ubiquitin‐protein ligase MARCH6, the immunity, inflammation, and growth‐related transcription factor HIVEP3, the biotin recycling enzyme BTD, the nucleo‐cytoskeletal anchor SYNE1, and the mRNA polyadenylation promoter SYMPK, and plasma BTD activity was elevated in iSGS patients compared with controls.^122^ Small RNA sequencing was performed on samples from four patients with traumatic tracheal stenosis and four normal control cases, 24 miRNAs with significant differential expression were identified, of which 13 were upregulated and 11 were downregulated, QRT‐PCR confirmed that miR21‐5p and miR214‐3p were upregulated, while miR141‐3p and miR29b‐3p were downregulated.^123^ In addition, miR21‐5p was shown to be downregulated by pirfenidone, thereby inhibiting fibroblast proliferation in the treatment of acquired tracheal stenosis.^124^

#### 3.7.2 Hypoxia

Hypoxic concentration ventilation or mechanical compression ischemia will lead to microenvironment hypoxia, which will further promote the transition of fibroblasts to myofibroblasts, and synthesize a large amount of ECM, causing fibrotic scars and stenosis. A study showed that hypoxic ventilation can aggravate laryngeal injury associated with endotracheal (ET) intubation in porcine animal model.^125^ Moreover, a study have shown that HIF‐1 α is involved in the pathogenesis of proximal tracheal stenosis (PTS) and may be a potential key regulator in the initiation and facilitation of this process.^126^ Another study showed that normal laryngotracheal fibroblasts can transdifferentiate into myofibroblast phenotypes under hypoxia in vitro, which may be mediated by the fibrosis‐related cytokine IL‐6.^84^ The mechanism of hypoxia in the development of LTS still needs further exploration.

#### 3.7.3 Autophagy

Tracheal stenosis may be ameliorated by enhancing the autophagic process. A study reported that the expression of autophagy is decreased in rabbit model of trachea stenosis, and low dose of erythromycin could increase the expression of autophagy and reduce the tracheal mucosal fibrosis.^127^ In addition, quercetin can also activate autophagy activity, thereby exerting antifibrotic activity.^42^

#### 3.7.4 Cell cycle

By arresting the fibroblast cell cycle, it may have therapeutic significance for tracheal stenosis. It has been reported β‐elemene inhibits the ILK/Akt pathway through the MIR143HG/miR‐1275/ILK axis, induces cell cycle arrest and apoptosis of airway granulation fibroblasts, thereby inhibiting airway granulation proliferation and ultimately alleviating AS.^128^ Dieckol, a phlorotannin derivative isolated from Ecklonia cava, induces cell cycle arrest by downregulating CDK2/cyclin E in response to p21/p53 activation in human tracheal fibroblasts.^129^

## 4 PRECLINICAL THERAPIES

The above discussion introduces the pathogenesis of LTS, which is representative of AS. Given the scarcity of research on the mechanisms of AS beyond LTS, and considering that various types of AS, as inflammatory fibrotic diseases, share certain commonalities in their mechanisms, the mechanism of LTS and existing preclinical studies can serve as a representative model for preclinical research on AS. To date, fibrosis, inflammation, EMT, metabolic reprogramming, microbiome, genetics, and other mechanisms have been proven to play a pivotal role in LTS. Various molecules and signaling pathways are crucial within these mechanisms, so targeting these molecules and signaling pathways can potentially alleviate the disease (Table 2).

**TABLE 2:** Preclinical therapies in airway stenosis.

Therapeutic strategy	Intervention	Targeting pathway/molecule	Diseases (model)	Author/year
Drug therapy	1D11 (TGF‐βantibody)	TGF‐β/Smads	TS (canine)	Simpson et al. 2008^130^
	p17 (TGF‐β antagonist)	TGF‐β/Smads	TS (rabbit)	Antón‐Pacheco et al. 2017^131^
	SB525334 (TβRI inhibitor)	TGF‐β/Smads	SGS (human)	Ghavimi et al. 2024^132^
	Nintedanib	TGF‐β/Smads, ERK	TS (rat)	Fan et al. 2021^133^
	Phlorotannin	TGF‐β/Smads, MAPK	GS (rabbit)	Kim et al. 2020^15^
		TGF‐β/Smads	TS (rabbit)	Lee et al. 2020^16^
	Phlorofucofuroeckol A	TGF‐β/Smads, MAPK	TS (human)	Heo et al. 2020^135^
	Plumbagin	TGF‐β/Smads, Akt/mTOR	TS (rat)	Shi et al. 2021^41^
	Pirfenidone	TGF‐β/Smads	GS (ferret)	Kodama et al. 2018^137^
		miR‐21‐5p	TS (human)	Li et al. 2024^124^
	Tanshinone IIA	TGF‐β/Smads, ZEB1, Snail1	TS (rat)	Duan et al. 2016^97^
	Triamcinolone	TGF‐β/CCN2/MMP9	SGS (human)	Treviño‐Villarreal et al. 2021^53^
	Quercetin	TGF‐β/AKT/mTOR	TS (rabbit)	Xiao et al. 2020^42^
	Rapamycin	mTOR	LTS (mouse)	Motz et al. 2023^79^
	MEK inhibitors	ERK	TS (mouse)	Kimura et al. 2021^155^
	β‐Elemene	Wnt/β‐catenin	TS (human)	Xue et al. 2018^165^
		MIR143HG	AS (rabbit)	Zhang et al. 2021^128^
	Erythromycin	NF‐κB	TS (rabbit)	Huang et al. 2021^166^
	Doxycycline	Unknown	TS (rabbit)	Choi et al. 2018^170^
	Anakinra	IL‐1R	LTS (mouse)	Nicolli et al. 2016^174^
	DON	Glutamine	LTS (human)	Murphy et al. 2018^100^
			LTS (mouse)	Tsai et al. 2021^102^
	BPTES	Glutaminase	LTS (human)	Tsai et al. 2020^101^
	Antimicrobial peptide	Unknown	LTS (mouse)	Aronson et al. 2023^186^
	Dieckol	CDK2/cyclin E	TS (human)	Heo et al. 2022^129^
	5‐fluorouracil	Unknown	LTS (rabbit)	Mao et al. 2017^188^
	Paclitaxel	Unknown	TS (canine)	Wang et al. 2016^189^
	Cisplatin	TGF‐β/Smads	GS (human)	Xu et al. 2020^190^
	CM‐chitosan	Unknown	TS (rabbit)	Huang et al. 2024^191^
Gene therapy	shGATA6‐LV	Wnt/β‐catenin, NLRP3	TS (rat)	Li et al. 2023^47^, ^69^
	Ad‐shRNA‐SOX9 LV‐shRNA‐SOX9	Wnt/β‐catenin	TS (rat)	Gu et al. 2022^48^
	CXCR7 siRNA	NF‐κB	LTS (human)	Xu et al. 2023^86^
	shRNA‐Robo1	TGF‐β/Smads	TS (rat)	He et al. 2023^175^
	LV‐shRNA‐IL‐11Rα	TGF‐β/Smads	TS (rat)	Xiao et al. 2023^85^
	FIR‐SeV/ΔF	c‐myc	TS (rat)	Mizokami et al. 2015^193^
Stem cell therapy	hAMSCs	Unknown	SGS (rat)	Oh et al. 2018^178^
	BMSCs	Unknown	LTS (canine)	Iravani et al. 2017^179^
	Autologous airway basal cell	Unknown	TS (canine)	Ye et al. 2023^180^
Growth factor therapy	GDF15	TGF‐β/Smads	TS (rat)	Liao et al. 2022^134^
	ARF	Unknown	TS (canine)	Chen et al. 2024^194^
Protein therapy	Recombinant Slit2	TGF‐β/Smads	TS (rat)	He et al. 2024^136^
Photodynamic therapy	PDT	Unknown	AS (rabbit)	Nakagishi et al. 2008^192^

AS, airway stenosis; LTS, laryngotracheal stenosis; GS, glottic stenosis; SGS, subglottic stenosis; TS, tracheal stenosis, shGATA6‐LV, lentiviruses containing short hairpin RNAs targeting GATA6; LV, lentiviral vector; Ad, adenoviral virus; FIR‐SeV/ΔF, sendai virus encoded far interacting repressor; hAMSCs, human adipose tissue‐derived mesenchymal stem cells; BMSCs, bone marrow stem cells; ARF, autologous regeneration factor.

### 4.1 Antifibrotic therapy

The fibrosis‐related signaling pathways of LTS primarily involve the TGF‐β and Wnt/β‐catenin signaling pathways. Targeting molecules within these pathways can yield significant therapeutic benefits (Figure 1). Although abnormal remodeling of the ECM is also a fibrotic mechanism, research on targeted therapy in LTS remains scarce.

#### 4.1.1 Targeting the canonical TGF‐β signaling for antifibrosis

Given the lack of research on targeting BMP signaling pathway to treat LTS, we will focus on TGF‐β/Smads signaling pathway. After a thorough study and understanding of the TGF‐β/Smads signaling pathway, several therapeutic strategies have been identified for LTS by targeting components within this pathway. They can be divided into five categories: Inhibition of TGF‐β and TβR, inhibition of Smad2/3 phosphorylation, inhibition of nuclear translocation, inhibition of the transcriptional activity of Smad complexes, and enhancement of I‐Smad expression.

##### Inhibition of TGF‐β and TβR

The TGF‐β antagonist has been tested in animal models with limited results. Antibody (1D11), which antagonizes TGF‐β, can sightly improve the tracheal stenosis and prolong survival time in canine model of LTS induced by cautery injury.^130^ In rabbits model of LTS induced by circumferential thermal injury, TGF‐β peptide antagonist (p17) significantly reduced fibrotic thickness and density of myofibroblasts, but failed to improve luminal narrowing.^131^ Although anti‐TGF‐β therapy is theoretically expected to have a significant impact, evidence to date indicates that there are substantial concerns about efficacy, safety, and off‐target effects, so targeting downstream of TGF‐β may be a more promising strategy.^5^ As for TGF‐β receptors, recent studies have shown that SB525334, an ALK5 (TβRI) inhibitor, has the superior potential for subglottic stenosis treatment by simultaneously modulating TGF‐β signaling pathway.^132^

##### Inhibition of Smad2/3 phosphorylation

When the bioactive TGF‐β ligands binding to the TβR complex, inhibiting the phosphorylation of Smad2/3 can prevent signal propagation, thereby inhibiting subsequent biological effects. Nintedanib was found to effectively prevent tracheal stenosis in rats by inhibiting fibrosis and inflammation, with a potential mechanism of inhibiting Smad2/3 phosphorylation.^133^ Another rat model of tracheal stenosis study showed that GDF15 supplementation alleviated the degree of tracheal stenosis, and GDF15 inhibited fibroblast activation via inhibiting Smad2/3 phosphorylation, thereby blocking the TGFβ1–Smad2/3 pathway.^134^ Phlorotannins, the polyphenolic compounds extracted from Ecklonia cava, suppress the expression of fibrosis phenotype markers by inhibiting the Smad2/3 phosphorylation in fibroblasts, showing efficacy in rabbit model of vocal fold fibrosis and tracheal stenosis.^15^, ^16^ In addition, a study proves it is plorofucofuroeckol A, an effective component in phlorotannins that plays the critical role.^135^ A recent study found that Slit2 supplementation and Robot1 downregulation can inhibit excessive ECM deposition induced by TGF‐β1 in RTFs via TGF‐β1/Smad3 pathway, and can attenuate airway fibrosis in a trauma‐induced rat airway obstruction model.^136^ Research on a similarly designed rat model demonstrates that plumbagin regulates fibroblast activity by decreasing the phosphorylation of Smad2/3 in vivo and vitro, which ultimately improves tracheal stenosis.^41^

##### Inhibition of nuclear translocation

Inhibiting the nuclear translocation of the trimeric complex containing Smad4 and p‐Smad2/3 can theoretically affect this pathway and its subsequent profibrotic gene expression. Studies have demonstrated that pirfenidone suppresses the translocation of p‐Smad2/3 from cytoplasm to nucleus in fibroblasts isolated from scarred vocal folds of ferrets.^137^ In other fibrotic diseases, N‐butyldeoxynojirimycin (miglustat), an inhibitor of glucosylceramide synthase (GCS) exerts its therapeutic effect on pulmonary fibrosis through inhibition of nuclear translocation of Smad2/3 rather than through suppression of TGF‐β1‐induced Smad2/3 phosphorylation.^138^ Fibroblast growth factor 21 (FGF21) attenuated nuclear translocation of Smad2/3 by inhibiting renal activity of its conjugated protein p53, which carries Smad2/3 into the nucleus, thus negatively regulating the TGF‐β/Smad2/3‐mediated EMT process and preventing renal fibrosis.^139^ Parthenolide might treat peritoneal dialysis‐related peritoneal fibrosis through inhibiting both TGF‐β‐induced Smad2/3 phosphorylation and nuclear translocation.^140^

##### Inhibition of the transcriptional activity of Smad complexes

After being recruited by the Smad trimeric complexes, the coactivators p300/CBP acetylates Smad2 and Smad3, acting on Lys‐19 in MH1 domain of Smad2 and Lys‐378 in the MH2 domain of Smad3, to change their conformation and facilitate DNA binding, thereby enhancing transcriptional activity.^141^, ^142^ Therefore, inhibiting the coactivator‐mediated acetylation, deacetylating Smads or other ways to ultimately block the binding of Smad complexes to DNA have therapeutic effects. In a study on the treatment of iSGS by subglottic serial intralesional steroid injections (SILSIs), triamcinolone, a steroid used in SILSIs showing good clinical efficacy in iSGS, impaired Smad2/3 binding to promoter regions and prevented the coregulation of CCN2 and MMP9 mRNA Expression induced by TGF‐β1 in iSGS primary fibroblasts.^53^ Curcumin, a acetyltransferase p300 inhibitor, can reduce the senescence ratio of atrial fibroblasts, ameliorate the atrial fibrosis and decrease the atrial fibrillation inducibility in aging mouse and senescence human through p53/Smad3 pathway.^143^ In the study of cardiac fibrosis, beraprost not only significantly reduced TGF‐β expression, Smad2 phosphorylation, and Smad‐DNA binding activity, but also increased phosphorylation of CREB at Ser133 and decreased Smad2 binding to CBP in the nucleus, which ultimately inhibited cardiac fibroblast proliferation by suppressing TGF β/Smad signaling pathway.^144^ Both SIRT1 and SIRT2 are nicotinamide adenine dinucleotide (NAD) dependent protein deacetylases, which can deacetylate Smad to improve renal fibrosis, and Sirt1 can be activated by resveratrol.^145^, ^146^ Death‐associated protein 6 (Daxx) interfered with Smad2 acetylation to reduce the transcriptional activity of Smad2, alleviating liver fibrosis in a thioacetamide‐induced fibrosis mouse model.^147^

##### Enhancement of I‐Smad expression

Inhibitory Smads, which inhibit TGF‐β signaling through multiple mechanisms, have shown good efficacy in keloids, diabetic kidney disease (DKD), liver cirrhosis, and myocardial infarction,^148^, ^149^, ^150^, ^151^ and may be a potential target of LTS. Ginsenoside Rg3 increased the expression of Smad7, which significantly inhibited the proliferation, migration, invasion, angiogenesis and collagen synthesis of human keloid fibroblasts (KFs) and suppressed angiogenesis and collagen accumulation in keloids through an ex vivo assay.^148^ A study on DKD suggested that latent TGF‐β1 may protect kidneys from TGF‐β1/Smad‐mediated renal fibrosis via inhibiting Arkadia‐mediated Smad7 ubiquitin degradation.^149^ Smad7 induction in myofibroblasts inhibited postinfarction fibrosis by restraining canonical and noncanonical TGF‐β responses, and by suppressing TGF‐β‐independent fibrogenic actions of ErbB2.^150^ The study on effectively treating liver fibrosis in the CCL_4_‐induced liver cirrhosis model by using bone marrow mesenchymal stem cells (MSCs) that overexpressed the Smad7 gene demonstrated that inhibition of TGF‐β1 signaling pathway by enhancement of Smad‐7 expression could be a feasible cell therapy approach to mitigate liver cirrhosis,^151^ which is also worth testing in LTS as a potential therapy

#### 4.1.2 Targeting the noncanonical TGF‐β signaling for antifibrosis

Although targeting the canonical TGF‐β signaling does show considerable therapeutic efficacy in treatment of LTS, there are also promising prospects in targeting noncanonical TGF‐β signaling pathway, including MPAK (ERK, JNK, p38) pathways and PI3K/Akt/mTOR pathway.

##### TGF‐β/Akt/mTOR pathway

Since the study showed that canonical PI3K/Akt signaling pathway is dispensable for TGF‐β1 stimulated collagen synthesis in human lung fibroblasts, it suggests that TGF‐β/mTOR signaling may play a dominant role, and 4E‐BP1 rather than p70S6K is the critical downstream of mTORC1 signaling.^152^ Additionally, plumbagin and quercetin can inhibit TGF‐β/Akt/mTOR signaling pathway in vivo and in vitro, thereby improving fibrosis.^41^, ^42^ In addition to inhibiting the proliferation, migration, metabolism, and function and promoting apoptosis of LTS‐derived fibroblasts, rapamycin targeting mTOR can effectively treat LTS through inhibition pf profibrotic Th17 cells.^79^, ^153^, ^154^

##### MAPKs pathways

Extracted from Ecklonia cava, phlorotannins and its active substance, Phlorofucofuroeckol A, can inhibit MAPKs signaling pathways, in addition to restraining TGF‐β/Smad signaling pathways.^15^, ^135^ As part of the MAPKs signaling pathway, ERK has been found to have therapeutic significances as a target in several studies. One of the therapeutic effects of nintedanib on tracheal stenosis and antifibrotic effect may be achieved through inhibiting the ERK1/2 signaling pathways.^133^ In a novel cauterization‐induced tracheal stenosis mouse model, inhibition of ERK phosphorylation by using MEK inhibitors, an upstream kinase of ERK, can improve tracheal stenosis.^155^ Besides inhibiting TGF‐β/Smad signaling pathway, ginsenoside Rg3 can also suppress the ERK signaling pathway, which ultimately inhibiting the proliferation, angiogenesis, and collagen synthesis of KF in vitro.^148^

#### 4.1.3 Potential targets for latent TGF‐β activation

Although there is no research on latent TGF‐β action in LTS, this gap can be addressed by drawing on the research experience of other fibrotic diseases. At present, there are potential molecular targets by inhibiting activation of latent TGF‐β, including TSP1, GAPR, recombinant truncated LAP, and integrins.

##### TSP1, LAP, and GAPR

Targeting TSP1 with miR‐221 prevents latent TGF‐β1 activation, thereby alleviating kidney failure‐induced cardiac fibrosis.^156^ In addition, blocking TSP1 can also mitigate renal interstitial fibrosis through the TSP1/CD47 signaling pathway, and ameliorate high glucose‐induced peritoneal fibrosis via downregulation of TGF‐β1/Smad3 signaling pathway.^157^, ^158^ The inhibition of TGF‐β1 activation by enhancing LAP may attenuate fibrosis, which has been demonstrated by a study showing that LAP and truncated LAP could alleviate liver fibrosis in vitro and in vivo via inhibition of TGF‐β/Smad signaling pathway.^159^ GARP expressed on HSCs drives the development of liver fibrosis via cell contraction‐mediated activation of latent TGF‐β, suggesting GARP as a novel target for the treatment of fibrotic disease.^160^

##### Integrins

To date, the activation mechanism of αv‐containing subset of integrins has been studied extensively. Therapeutic delivery of αvβ1 inhibitor, compound 8 (c8) inhibits αvβ1 integrin‐mediated TGF‐β activation, attenuating bleomycin‐induced pulmonary fibrosis and carbon tetrachloride‐induced liver fibrosis.^161^ GSK3008348 can alleviate murine bleomycin‐induced pulmonary fibrosis by inhibiting αvβ6 integrin, highlighting the potential of inhaled GSK3008348 as an antifibrotic therapy.^162^ ADWA‐11, a αvβ8 integrin function‐blocking antibody, ameliorated both canonical TGF‐β signaling pathway and lens epithelial cell fibrotic response, which ultimately preventing posterior capsular opacification.^163^

#### 4.1.4 Targeting the Wnt/β‐catenin signaling for antifibrosis

Since the Wnt/β‐catenin signaling pathway plays crucial role in LTS, targeting components of the pathway may be an effective strategy (Figure 2). Here, we summarize the existing treatments for LTS through inhibiting Wnt/β‐catenin. β‐Elemene has been shown to induce apoptosis and necrosis of airway primary fibroblasts and inhibit proliferation of fibroblasts and airway granulation through upregulating the activity of GSK3β and suppressing protein level and nuclear translocation of β‐catenin.^164^, ^165^ In addition, other studies found that GATA6 silencing and SOX9 knockdown can also enhance the activity of GSK3β and inhibit nuclear translocation and transcriptional activity of β‐catenin, which ameliorates injury‐induced tracheal fibrosis in vivo and in vitro.^47^, ^48^

### 4.2 Anti‐inflammatory therapy

The NF‐κB signaling pathway and immune microenvironment dysregulation are important mechanisms by which inflammation leads to LTS. Targeting these pathways and molecules can produce good therapeutic effects. Unfortunately, there are few studies on targeted treatment of LTS in this field. In addition, some anti‐inflammatory drugs have shown certain efficacy in the application of LTS.^166^

It has a therapeutic effect by targeting the NF‐κB signaling pathway (Figure 3). Current research mainly focuses on inhibiting NF‐κB binding to DNA and its transcriptional activity. Erythromycin can upregulate the expression of HDAC2, which alleviates the inflammation of trauma‐induced TS and changes the progression of fibrosis by inhibiting the transcription of NF‐κB.^166^, ^167^, ^168^, ^169^ In addition, another anti‐inflammatory drug, doxycycline, has a good effect on reducing inflammation and fibrosis in rabbit models when loaded with nitinol stents.^170^ Glucocorticoids can inhibit the DNA‐binding activity of NF‐κB, and serial in‐office intralesional steroid injection has shown to be safe and well‐tolerated in adults with SGS or PTS.^8^, ^68^

Targeting NLRP3‐related pathway has potential therapeutic significance. A study has demonstrated that downregulation of GATA6 alleviates NLRP3 inflammasome‐mediated pyroptosis induced by tracheal injury in rats, thereby alleviating tracheal stenosis, inflammation, and fibrosis.^69^ Moreover, NLRP3 inhibitors MCC950, oridonin, and tranilast have preventive or therapeutic effects in a variety of inflammatory diseases and are worth trying in the drug treatment of LTS.^171^, ^172^, ^173^ IL‐1 targeted agents, anakinra, rilonacept, and canakinumab can treat inflammation in a variety of inflammatory diseases, and anakinra can prevent early granulation formation in LTS.^173^, ^174^

### 4.3 Anti‐EMT therapy

The process of EMT can generate fibroblasts, which are then activated into myofibroblasts to produce a large amount of collagen, leading to ECM accumulation and ultimately fibrosis, clinically manifested as LTS. By inhibiting the signaling pathways and molecules involved in the EMT process, good therapeutic effects can be demonstrated.

Recombinant GDF15 weakly promotes EMT but attenuates TGFβ1‐induced EMT in tracheal epithelial cells.^134^ Tanshinone IIA alleviates EMT in vivo and in vitro by inhibiting the Smad signaling pathway and the expression of transcription factors ZEB1 and Snail1, thereby reducing tracheal stenosis after tracheal transplantation.^97^ Silencing Robo1 inhibits Smad3 and thereby attenuates EMT in tracheobronchial stenosis.^175^ CXCR7 activation promotes the migration of LTS‐derived fibroblasts by inhibiting E‐cadherin expression through the NF‐κB signaling pathway.^86^ GATA6 overexpression promotes EMT in tracheal epithelial cells by regulating the NF‐κB/NLRP3 pathway.^69^ IL‐11 promotes EMT of tracheal epithelial cells by activating β‐catenin signaling.^85^

### 4.4 Targeting fibroblast metabolism therapy

By targeting the aerobic glycolysis, glutamine catabolism, and oxidative phosphorylation of fibroblasts with abnormal metabolism, it may have potential therapeutic value. Rapamycin significantly reduces oxidative phosphorylation in LTS fibroblasts, and its antifibroblastic effects suggest it is a promising adjunctive therapy for the treatment of LTS.^153^ The glutamine antagonist DON reverses profibrotic changes by inhibiting glycolysis and oxidative phosphorylation of iLTS scar fibroblasts, and significantly reduces fibrosis in iLTS mice.^100^, ^102^ In addition, the glutaminase inhibitor BPTES can significantly inhibit the proliferation and function of iLTS scar fibroblasts.^101^

### 4.5 Stem cell therapy

Stem cell therapy is currently the most important research area for AS, especially MSCs, which have shown some therapeutic effects by being injected into the scar area or used as components of tissue engineering.^176^ MSCs can be derived from bone marrow, fat, synovial membranes, and umbilical cord, and one of their main effects is to regulate inflammation and immune response to tissue injury,^177^ which has good prospects for the treatment of AS.

Good therapeutic effect was shown by injecting stem cells into the lesion. A study have shown that injecting human adipose tissue‐derived MSCs (hAMSCs) can help prevent subglottic stenosis in rats.^178^ Another study showed that local injection of bone marrow stem cells (BMSCs) can improve the LTS in dogs.^179^ In addition, by transplanting autologous airway basal cells, the defective airway epithelium can be repaired, thereby inhibiting recurrent granulation hyperplasia in canine benign tracheal stenosis.^180^

Tissue engineering with stem cell‐filled scaffolds for AS. Using stem cell‐filled scaffolds to create tracheal replacements shows promise for treating AS.^181^ In vivo tissue‐engineered tracheal regeneration by seeding MSCs in decellularized matrices may be a potential therapy for tracheal injury.^182^, ^183^ This technology has been applied clinically, by inoculating bone marrow MSCs into decellularized tracheal scaffolds and implanting autologous epithelium to form tracheal substitutes and replace the tracheal stenosis segments, successfully treating a 12‐year‐old boy with congenital tracheal stenosis and pulmonary sling.^184^

### 4.6 Other preclinical therapies

For the microbiome, by regulating the respiratory microbiota, it can affect the local microenvironment and achieve therapeutic effects. A novel drug‐eluting ET tube for delivering model the antimicrobial peptide Lasioglossin‐III (Lasio) has been engineered, which can regulate the airway microbiome during intubation, thereby reducing T cell and macrophage responses and reducing SGS in vivo.^185^, ^186^ For the cell cycle, both β‐elemene and Dieckol can induce cell cycle arrest in fibroblasts, thereby reducing their activation and secreting collagen leading to ECM accumulation.^128^, ^129^ Some antitumor biologics have also been applied to LTS. Given the similarity in metabolism between abnormal fibroblasts and tumor cells, known as the Warburg's effect, this method has shown certain therapeutic effects. 5‐FU encapsulated ethosomes are effective for LTS in rabbit models, and topic administration of 5‐FU ethosomes may be a novel candidate therapy for LTS treatment.^187^, ^188^ Paclitaxel eluting tracheal stent can reduce granulation tissue formation in a canine model.^189^ Cisplatin can prevent postoperative vocal cord scarring and laryngeal stenosis in patients undergoing CO_2_ laser microsurgery and delayed wound healing.^190^ In addition, a study using nebulized administration showed that inhaling CM chitosan can alleviate post traumatic tracheal fibrosis in rabbit models, providing a potential new treatment for tracheal stenosis.^191^ Photodynamic therapy (PDT) is effective for AS in rabbit models and has the potential to as a new therapeutic method for the caused by granulation tissue.^192^ A study has shown that sendai virus encoded far interacting repressor (FIR‐SeV/Δ F) can prevent tracheal stenosis in a rat model of airway mucosal injury by inhibiting the expression of c‐myc in the tracheal mucosa.^193^ A recent study found that autologous regeneration factor (ARF) facilitates tracheal mucosal wound repair and ameliorates tracheal fibrosis to improve BAS.^194^

Preclinical research on AS is quite rich and prosperous, and has shown the prospect of overcoming AS, but there is still one study that has been successfully clinically transformed. In contrast, existing clinical treatments, including endoscopic treatment and surgical treatment, are not ideal. These treatments not only bring pain to patients, but also patients still have to face the risk of recurrence and related functional impairment.

## 5 CLINICAL MANAGEMENT OF THE AS

AS usually involves supraglottic, glottic, subglottic, and trachea stenosis, that is, LTS. Therefore, the current clinical management of AS is mainly aimed at LTS, and the clinical management in this field is relatively systematic. However, the management of other sites of the AS is more scattered. Considering that its pathogenesis is similar to LTS, there are some common points in clinical management. The clinical management of AS include surgical management and nonsurgical management^195^; surgical management covers endoscopic airway surgery and open airway surgery, while nonsurgical management includes drug therapy, gas therapy and radiotherapy.

### 5.1 Endoscopic airway surgery

As a minimally invasive and low‐risk surgical option, endoscopic interventions can often be performed safely in outpatient procedures while helping to maintain good voice and swallowing function.^196^ However, this approach does come with a higher recurrence rate and the risk of reoperation. It is important to note that repeated endoscopic treatments can cause mucosal damage, granuloma overgrowth, bleeding, and tearing, which can eventually lead to the risk of disease recurrence or even asphyxia.^197^ The core strategy of endoscopic surgery lies in scar disruption (resection or lysis) and dilation. Scar resection is accomplished through the use of laser, cold steel and electrocautery, while scar lysis is facilitated by cryotherapy and radiofrequency coblation. Dilation is achieved by balloon or rigid dilation and stent implantation. These treatments can be used individually or in combination.

#### 5.1.1 Laser, cold steel, and electrocautery

Scar resection is usually performed by making three to four radial incisions in the area of stenosis.^198^ If three incisions are made, they are usually placed at the 12, 4, and 8 o'clock according to anatomy. If four incisions are made, they are located at the 12, 3, 9, and 6 o'clock.

Currently, the most widely used laser is the carbon dioxide laser. Endoscopic CO_2_ laser therapy has demonstrated excellent efficacy in patients with Myer‐Cotton grade I and II AS. Additionally, CO_2_ laser scar resection is often performed in conjunction with dilation and/or medication‐assisted therapy. A study involving 19 patients with SGS indicated that endoscopic CO_2_ laser, combined with balloon dilation and steroid injection, represents a safe, reliable, and minimally invasive endoscopic treatment approach.^199^ Another study, which surveyed 810 patients with idiopathic SGS, revealed that in the use of adjuvant therapy after CO_2_ laser resection, the recurrence rate of the fully compliant group was lower than that of the partially compliant group and the completely noncompliant group.^200^ A few cases have also reported the use of Nd:YAG laser and blue laser. A study showed that Nd:YAG laser as a traditional way combined with rigid bronchoscopic dilation can be used as an initial treatment for concentric tracheal stenosis.^201^ Recently, the blue laser has been applied in the treatment of laryngeal stenosis. When compared with the control group represented by the CO_2_ laser, there are no significant differences in terms of objective surgical score, stenosis recurrence rate, and complication rate, indicating a certain degree of therapeutic efficacy.^202^ Cold steel is gradually replaced by CO_2_ laser due to its higher risk of multiple operations, especially for patients who require repeated sugery.^203^ In addition, another study showed that electrocautery needle knife combined with balloon dilation in the treatment of benign tracheal stenosis can significantly improve the airway mucosal injury severity and fibrous tissue hyperplasia.^204^

#### 5.1.2 Cryotherapy and radiofrequency coblation

Cryoablation employs cryogens such as NO, CO_2_, or liquid nitrogen to repeat the freeze–thaw cycle, either to induce tissue necrosis or to freeze tissue for immediate removal with forceps.^205^ While bipolar radiofrequency plasma ablation, which is commonly used in orthopedics and otolaryngology surgery, generates a local high‐energy plasma field that can simultaneously ablate tissue and seal blood vessels.^206^ Both of these methods can make scar tissue necrosis and realize scar lysis, reducing the risk of fire compared with the working temperatures of 400–600°C for monopolar cautery and CO_2_ lasers.^207^, ^208^ Multiple studies have demonstrated that cryotherapy is a safe and effective treatment for LTS, and it can be used in conjunction with balloon dilatation.^208^, ^209^, ^210^ Similarly, numerous studies have confirmed that radiofrequency ablation is an effective surgical method for treating AS in both pediatrics and adults.^206^, ^211^, ^212^

#### 5.1.3 Dilation and stent implantation

Dilation is another type of AS, mainly through rigid dilation represented by rigid bronchoscope, balloon dilation, and stent implantation. The dilation strategy is primarily suitable for patients with simple AS and inoperable complex AS. Initially, a rigid bronchoscope is used for initial dilation, followed by the application of a balloon when deemed appropriate. For patients with inoperable complex AS, if the aforementioned methods fail, stent implantation may be considered.^213^

A retrospective cohort study including 63 patients with subglottic stenosis and tracheal stenosis showed that balloon and rigid bronchoplasty are safe and effective endoscopic tools for the early treatment of benign subglottic stenosis and tracheal stenosis,^214^ and another retrospective study showed that compared with rigid dilation, balloon dilation had a slightly better long‐term effect.^215^ Balloon dilations are favored as they expand the stenotic area through measured and controlled radial pressure. In contrast, rigid dilators are sidelined because the shear forces applied in the stenotic sector hypothetically cause mucosal injury, resulting in fibrosis and worsening of the disease.^216^ However, studies have shown that rigid dilators are the primary treatment for LTS in pediatrics, and rigid dilation is a relatively cheap and effective tool whose success rate is in the same range as balloon dilation.^216^, ^217^

In addition to balloon dilation, airway stents serve as another means of dilation. For patients with complex AS who are ineligible for surgery, the implantation of airway stents is the ultimate palliative treatment. Traditional stents primarily consist of two types: silicone stents and metal stents. Silicone stents are extensively utilized due to their ease of removal and repositioning, yet their drawbacks cannot be overlooked, including migration, granulation tissue formation, and airway obstruction.^218^ Conversely, metallic stents exhibit a lower migration rate and a greater airway cross‐sectional diameter (owing to the thinner wall construction), allowing them to better accommodate irregular airways, maintain epithelialization within the stent to preserve the mucociliary clearance function, and facilitate a simpler placement process.^218^ Nevertheless, metallic stents also pose certain complications, such as stent fracture, migration, granulation tissue formation, impaired mucociliary clearance, recurrent stent obstruction by the lumen, and increased bacterial infection.^218^ Montgomery T‐Tube, as a representative of silicone stent, has been proven in a retrospective cohort study involving 52 patients with tracheal stenosis to effectively alleviate their respiratory distress symptoms and enhance their quality of life with safety profile.^219^ The Dumon silicone stent, another type of straight silicone stent, is utilized for patients who have not undergone tracheotomy. Compared with the Montgomery T‐Tube, which is used for patients who have undergone tracheotomy, these two silicone stent exhibit low morbidity and excellent clinical outcomes in the treatment of patients with benign LTS, with a large proportion of patients free of airway instrumentation on long‐term follow‐up.^220^ A novel surgical approach, known as Maddern surgery, involves endoscopic excision of scar tissue and subsequent reconstruction of the mucosa by covering a silicone T‐tube with split‐thickness skin graft.^221^ Self‐expanding metal stents (SEMSs) are the most commonly used type of metal stents. In a retrospective cohort study, 116 patients with AS who underwent treatment with SEMSs were included. The study findings suggest that permanent SEMS treatment is an effective and safe option for most patients with benign tracheobronchial stenosis.^222^ Another study has demonstrated that third‐generation SEMSs represent a safe treatment option for complex BAS, but complications requiring stent removal are frequent.^222^

In the clinical practice of endoscopic treatment for AS, it not only assesses the severity of stenosis in the early stages and administers preliminary treatment, but also lays the groundwork for subsequent surgical procedures. For patients with Myer‐Cotton grades I and II, endoscopic treatment has demonstrated good efficacy and effectively prevents the need for further surgical intervention. However, for patients with Myer‐Cotton grades III and IV, as well as those who experience repeated recurrences after endoscopic treatment, open surgery appears to be a superior alternative.

### 5.2 Open airway surgery

Currently, the primary surgical procedures for AS include LTR, tracheal or cricotracheal resection with anastomosis (TRA or CTRA), and slide tracheoplasty (STP).^18^ Among these, TRA or CTRA is considered the gold standard for treating AS. LTR and STP are primarily utilized for pediatric congenital AS, which have recently been increasingly applied to adult AS cases.^223^, ^224^

#### 5.2.1 Laryngotracheal reconstruction

The method of LTR involves splitting the cricoid cartilage and transplanting cartilage to enlarge the lumen diameter, thereby improving the stenosis. The commonly chosen cartilages are costal and thyroid cartilages. Based on the site of cricoid cartilage split and the location of cartilage transplantation, LTR can be categorized into three types: LTR with anterior graft, LTR with posterior graft and LTR with anterior and posterior graft.^6^ Based on whether patients require tracheotomy and intubation postsurgery, LTR is further categorized into single‐stage LTR (SSLTR) and double‐stage LTR (DSLTR), both of which demonstrate favorable therapeutic outcomes. A retrospective cohort study involving 15 airway patients who underwent SSLTR demonstrated that for complex glottic‐subglottic stenosis, SSLTR can achieve long‐term airway patency, along with reasonable sound quality and normal swallowing.^225^ Another study indicated that DSLTR is also a safe and effective option for treating complete or severe LTS.^226^

#### 5.2.2 Tracheal or cricotracheal resection with anastomosis

For tracheal stenosis, trachea resection is chosen, whereas for subglottic stenosis, cricotracheal resection is opted for, followed by end‐to‐end anastomosis.^227^ Based on the anastomosis method, different types are classified: Type A involves the removal of only the tracheal rings, with subsequent tracheo‐tracheal (Type A1) or crico‐tracheal anastomosis (Type A2); Type B refers to the removal of the first tracheal rings with the cricoid arch and subsequent thyro‐crico‐tracheal anastomosis; Type C involves the excision of the anterior cricoid cartilage arch, resection of the surrounding mucosa at the cricoid cartilage level, reduction of the cricoid cartilage plate thickness using a burr, covering the area with the posterior wall of the trachea's mucosal flap, and then performing the thyro‐crico‐tracheal anastomosis; Type D involves the resection of the anterior cricoid cartilage arch, cleavage of the posterior cricoid cartilage to its upper edge, insertion of a costal graft, and then thyro‐crico‐tracheal anastomosis.^228^ Two multicenter studies have demonstrated that even for high‐grade LTS, TRA and CTRA exhibit extremely high success rates.^228^, ^229^ Additionally, the classification system of the European Laryngological Society can accurately predict the success rate of TRA/CTRA surgery in adults, potentially aiding in treatment selection and patient counseling.^229^ In addition, TRA represents an effective treatment in post‐COVID‐19 LTS patients.^230^ Moreover, TRA/CTRA yields favorable outcomes, characterized by rapid postoperative functional recovery, high quality of life for patients, and a good long‐term prognosis.^231^ However, factors such as traumatic stenosis, longer T‐tube duration, combined glottic/subglottic stenosis, start of stenosis at the level of vocal cords, postoperative minor complications, and need for repeat surgery may potentially impact the prognosis.^232^

#### 5.2.3 Slide tracheoplasty

STP, initially utilized for treating congenital tracheal stenosis, has now emerged as the primary treatment option for open management of tracheal stenosis. Following the determination of the stenosis scope under endoscopy, an incision is made at the midpoint of the stenosis, extending obliquely to the posterior wall of the trachea to achieve transection.^6^ The trachea is then trimmed, with the posterior surface of the distal end of the stenosis separated from the anterior surface of the proximal end until the stenotic segment is released.^6^ Subsequently, the tracheal segments are sutured together. A retrospective cohort study involving 80 pediatric patients with congenital tracheal stenosis who underwent STP demonstrated the effectiveness of STP in treating congenital tracheal stenosis.^233^ Additionally, another study indicated that STP treatment for CTS yields favorable long‐term outcomes.^234^ Furthermore, a study indicated that STP remains a viable reconstruction option for adult patients with tracheal stenosis, even for those with a history of previous airway reconstructions.^224^

### 5.3 Nonsurgical management

Currently, nonsurgical treatments for AS involve drug therapy, gas therapy, and radiotherapy.

Regarding drug therapy, there are only a handful of drugs currently employed in clinical practice. While numerous preclinical studies have confirmed the efficacy of certain drugs for treating AS, none of these drugs have undergone clinical trials or been approved for clinical use. The drugs currently in clinical use demonstrate certain effects, but they are not miracle cures and are subject to some controversy. Steroid can inhibit inflammation and fibroblast proliferation, while promoting collagen degradation. They are commonly used in patients with subglottic stenosis, and their administration routes include intralesional injection, nebulized inhalation, and systemic application, with intralesional injection being the most widely used. SILSI has been proven to be safe for the whole body, without accumulating systemic steroid side effects.^235^ It has been validated in multiple studies as effective for SGS, reducing the surgical burden on these patients and potentially avoiding future airway interventions, while significantly improving the patient's voice function.^8^, ^236^, ^237^ The effect of inhaled steroids on AS is controversial. One study demonstrated that Inhaled budesonide is effective for treatment of tracheal granulation tissue in patients with tracheostomies after repair of congenital tracheal stenosis.^238^ Conversely, another study indicated that inhaled steroids had no effect on patients with LTS after balloon dilatation.^239^ Studies on systemic application of steroids are scarce. Given their serious systemic side effects and toxic reactions, one study indicated that early low‐dose systemic corticosteroids are beneficial for the treatment of tracheal stenosis after intubation.^240^ Mitomycin C, an antitumor alkylating agent, can inhibit DNA and protein synthesis, and it can induce fibroblast apoptosis.^240^ Its curative effect on AS is also controversial. Studies have shown that mitomycin C can reduce the speed of subsequent granulation tissue and scar formation after endoscopic surgery, and it poses no side effects.^241^, ^242^ It is an effective drug for the adjuvant treatment of LTS. However, another study showed that mitomycin C, as a local adjuvant therapy, offered no additional benefit for endoscopic surgical treatment of LTS.^243^

The administration of immunomodulatory drugs to mitigate local airway inflammation theoretically reduces fibroblast migration and subsequent scar formation. This approach has been employed in autoimmune or vasculitic cases of LTS, such as in granulomatosis with polyangiitis (Wegener disease), typically using a combination of high‐dose corticosteroids with cyclophosphamide, rituximab, methotrexate, mycophenolate mofetil, or azothioprine.^9^ A study indicates that low‐dose methotrexate emerges as a novel and potentially beneficial adjunct therapy for patients with recurrent nonvasculitic LTS, potentially extending the interval between surgeries.^9^ Another recent study reveals that methotrexate and rituximab could serve as treatment options for some SGS patients experiencing a high recurrence rate.^244^

In addition, the use of triple‐drug adjuvant therapy (inhaled corticosteroids, proton pump inhibitors, and trimethoprim‐sulfamethoxazole) following endoscopic surgery has demonstrated significant efficacy.^200^

The principle of gas therapy is to reduce airway resistance and respiratory effort in patients with AS. Currently, hydrogen–oxygen mixture, as well as Heliox, have been applied to patients with AS.^245^, ^246^

Low‐dose radiotherapy could be a promising tool to prevent granulation tissue formation after surgery and/or endobronchial interventions regarding its established role in the treatment of keloids or hypertrophic scars, two benign diseases with similar a pathophysiology to tracheal stenosis.^247^ And a study has shown that low‐dose postoperative external beam radiotherapy is a new treatment for patients with recurrent tracheal stenosis who have failed endoscopic treatment, effectively reducing the frequency of endoscopic dilatation.^248^

## 6 CONCLUSIONS AND FUTURE PERSPECTIVES

AS poses a significant clinical challenge due to the narrowing of airway, leading to substantial morbidity and mortality. Understanding the classification of AS helps us choose surgery. For example, for AS of Myer‐Cotton grade III or above, we tend to choose open surgery rather than endoscopic surgery. Currently, the clinical treatments for AS are mainly endoscopic surgery and open surgery. The former mainly involves CO_2_ laser scar removal followed by balloon dilatation and drug‐assisted treatment, while the latter mainly involves tracheal or cricotracheal resection followed by end‐to‐end anastomosis. Both treatment methods have disadvantages and bring pain to patients. The high recurrence rate of endoscopic surgery and the sacrifice of swallowing and swallowing function by open surgery have promoted the development of new treatments. Developing novel management requires a better understanding of the pathogenesis of AS. Over the past few decades, researchers have developed a series of preclinical treatments based on the discovered mechanisms. These treatment strategies can be roughly divided into drug therapy, gene therapy, stem cell therapy, growth factor therapy, protein therapy, and PDT (Table 2).

**FIGURE 4:**
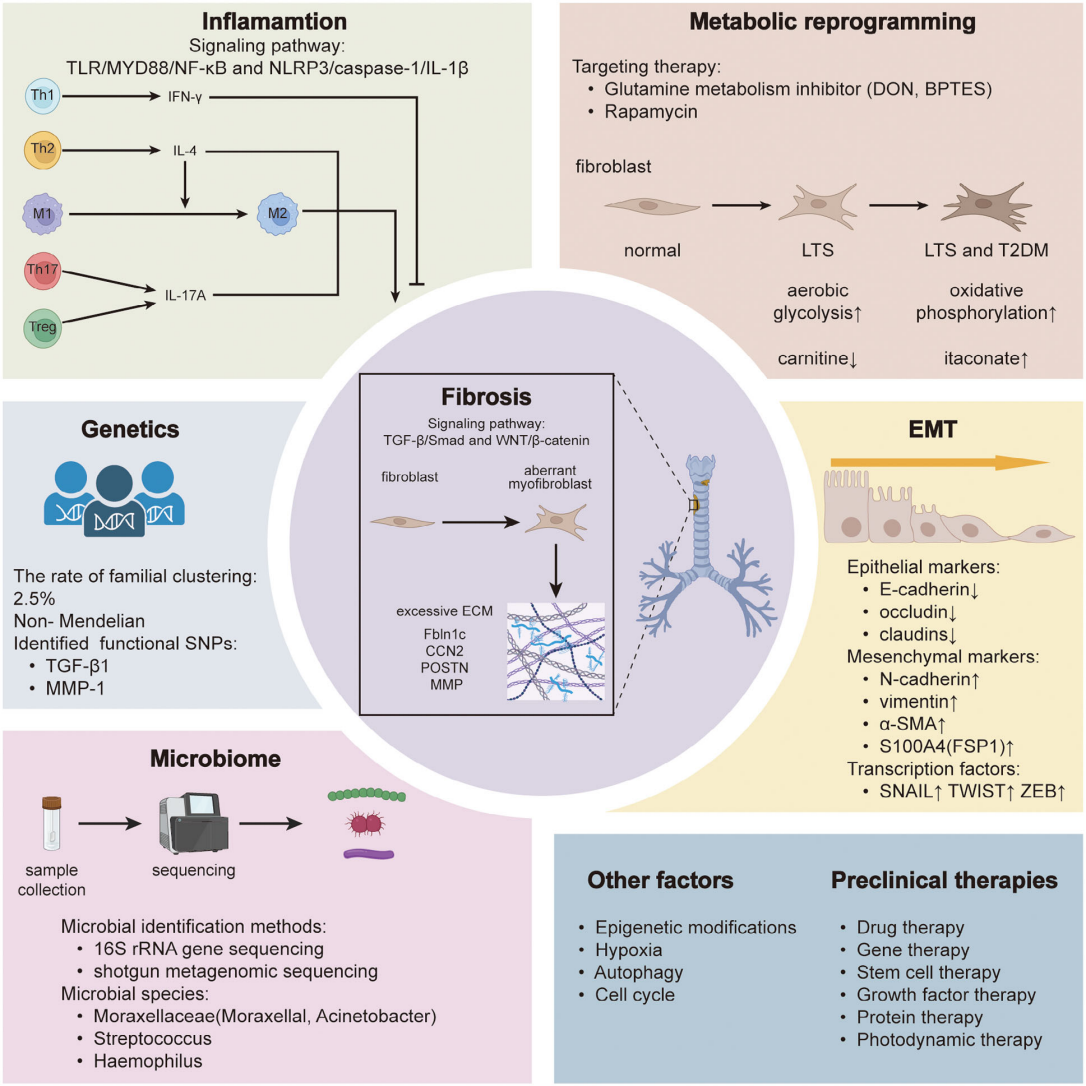
Mechanistic models of AS. Fibrosis is a core process that can be affected by other factors to accelerate or slow down the onset of AS, which is caused by abnormal fibroblasts synthesizing excessive ECM, Some ECM components, such as Fbln1c, CCN2, POSTN, MMP, have been found to affect the occurrence and development of AS. In terms of inflammation, inflammation‐related signaling pathways and several immune cells and their secreted cytokines can affect fibrosis. Metabolic reprogramming contributes to AS altering fibroblast metabolic characteristics and metabolites, and targeted metabolic drugs have curative effects. Genetic characteristics of AS, and the identified functional SNPs. EMT can produce fibroblasts and myofibroblasts, accompanied by a decrease in epithelial markers and an increase in mesenchymal markers and transcription factors. Microbial species associated with AS were identified by sequencing. Other factors, such as epigenetic modifications, hypoxia, autophagy, and cell cycle participate in the development and progress of AS. Many preclinical treatments have been developed based on these mechanisms and can be divided into drug therapy, gene therapy, stem cell therapy, growth factor therapy protein therapy, and photodynamic therapy.

The mechanisms of AS discovered so far mainly include fibrosis, inflammation, EMT, metabolic reprogramming, microbiome, and genetic susceptibility. These mechanisms do not act alone, but rather interact with each other and ultimately lead to the occurrence of stenosis (Figure 4). It would be beneficial to explore the crosstalk between different cell types and between the microorganisms and cells to identify the intersection of multiple mechanisms, which may be key to controlling the fibrotic process of the LTS and targeted treatment. The use of single‐cell RNA sequencing (scRNA‐seq) and spatial transcriptomics approaches may provide new mechanistic insights into the interactions between different cells. scRNA‐seq has been applied to the study of the mechanism of AS. A study identified a type of macrophage expressing S100A8/9 in iSGS patient specimens through scRNA‐seq. Therapy that inhibits the production of S100A8/9+ macrophages or S100A8/9 may be a beneficial treatment strategy for iSGS.^72^ With the help of scRNA‐seq, it was found that normal airway epithelial cells and basal cells were missing in AS.^94^ Transplantation of basal cells through combined tissue engineering methods can induce transplantable granulation proliferation.^180^ Regarding the interaction between microorganisms and cells, 16S rRNA gene sequencing and shotgun metagenomic sequencing can identify respiratory flora associated with AS, which may be pathogenic bacteria or probiotics. These flora can affect immune cells by producing metabolites, thereby producing subsequent biological effects. There are quite a few such studies in the field of intestinal flora. A study on pulmonary fibrosis identified three lung commensal microbes that can promote pulmonary fibrosis by inducing IL‐17B expression in alveolar macrophages through outer membrane vesicles and regulating the profibrotic inflammatory cytokine network.^249^

Future research should prioritize several specific areas to refine pathogenic theories and develop novel therapies. Signal transduction pathways, including the PI3K/Akt pathway, MAPK pathways (ERK1/2, JNK, and p38/MAPK signaling pathways), and Rho‐like signaling pathways from the TGF‐β signaling pathway, warrant deeper investigation to better understand noncanonical pathways in LTS pathogenesis. Exploring therapies targeting these pathways may lead to the development of potential therapeutic agents. Additionally, targeting the BMP signaling pathway could be explored in LTS, as no reports currently exist on its application in this condition. Preventive methods might also be more beneficial for patients with tracheostomy or prolonged intubation. The concept of using drug‐eluting stents for LTS prevention and treatment has attracted considerable interest among physicians and material scientists. Once a therapeutically potent agent is identified, it could potentially be integrated onto the cuff of an ET tube to prevent LTS after prolonged intubation or loaded onto a wet‐adhesive hydrogel to prevent LTS following trauma or iatrogenic injury. In clinical settings, large‐scale prospective cohort studies or clinical trials are needed to evaluate the advantages and disadvantages of various available treatment options such as balloon dilation, laryngoscopic surgery, and flap reconstruction surgery. This will enhance patient care until novel effective medications become available. Such efforts are crucial for improving clinical outcomes and quality of life for LTS patients in the near future.

In conclusion, AS is a complex clinical disease, and its mechanisms involve fibrosis, inflammation, EMT, metabolic reprogramming, microbiome, genetic susceptibility, and other mechanisms. The shortcomings of current clinical treatments have promoted the development of preclinical animal experiments, and a large number of interventions have shown objective effects, but there is still a certain distance from clinical trials and clinical use. Since we have comprehensively sorted out the preclinical treatment of AS, this information will contribute to the development and treatment of AS, as well as the future research direction of AS treatment, which will help explore and apply new treatment strategies.

## AUTHOR CONTRIBUTIONS

Pengwei Zhao and Zheng Jiang conceptualized this review. Pengwei Zhao, Zheng Jiang, and Xuexin Li drafted the manuscript. Pengwei Zhao is responsible for completing the figures and tables. Zheng Jiang and Pengwei Zhao revised the manuscript. All authors read and approved the final manuscript.

## CONFLICT OF INTEREST STATEMENT

The authors declare no conflicts of interest.

## ETHICS STATEMENT

Not applicable.

## Data Availability

Not applicable.
